# Identification of TIFY/JAZ family genes in *Solanum lycopersicum* and their regulation in response to abiotic stresses

**DOI:** 10.1371/journal.pone.0177381

**Published:** 2017-06-01

**Authors:** Andrea Chini, Walid Ben-Romdhane, Afif Hassairi, Mourad A. M. Aboul-Soud

**Affiliations:** 1 Plant Molecular Genetics Department, Centro Nacional de Biotecnología-CSIC (CNB-CSIC), Madrid, Spain; 2 Department of Plant Production, College of Food and Agricultural sciences, King Saud University, Riyadh, Kingdom of Saudi Arabia; 3 Centre of Biotechnology of Sfax (CBS), University of Sfax, LPAP, Sfax, Tunisia; 4 Department of Clinical Laboratory Sciences, College of Applied Medical Sciences, King Saud University, Riyadh, Kingdom of Saudi Arabia; 5 Biochemistry and Molecular Biology Department, Cairo University Research Park, Cairo University, Giza, Egypt; Instituto de Biologia Molecular y Celular de Plantas, SPAIN

## Abstract

Plant phenotypic plasticity determines plant adaptation to changing environments and agricultural productivity. Phytohormones are essential plant signalling molecules regulating this plasticity through complex signalling networks. Jasmonates (JAs) are key phytohormones regulating many aspects of growth, development and defence responses. An important role of JAs in tolerance to abiotic stresses is also emerging. The expression of *JAZ* (*JASMONATE-ZIM-DOMAIN PROTEIN*) genes, encoding for the key repressors in the JA-pathway, is regulated by multiple abiotic stresses, suggesting a role for the JAZ proteins in response to these stresses. The JAZ proteins belong to the TIFY family, well described in many plant species. However, only the role of few tomato JAZ proteins in response to microbial infection has been analysed so far. Here, we identify the members of the tomato *TIFY* family, and characterize them phylogenetically. In addition, we analyse the transcriptional regulation of several *SlJAZ* in response to abiotic stresses and hormone treatments both in root and leaves to assess their specific expression in response to stresses. Most *SlJAZ* are JA-induced and responsive to one or more abiotic stresses, providing clues for functional analysis of *JAZ* genes in abiotic responses in tomato.

## Introduction

Plants are sessile organisms that need to adapt to the ever-changing environment to survive and prosper. In addition, plants are constantly exposed to a myriad of different organisms such pathogens, pests or beneficial microorganisms that alter the plant-environment equilibrium. Endogenous and external inputs are integrated in complex signalling networks regulating plant-environment and plant-organism communications and, ultimately, plant survival. Plant hormones, or phytohormones, play a pivotal role in these signalling networks and regulate countless adaptive responses to both biotic and abiotic challenges. Traditionally, jasmonates (JAs) were described as defence signals regulating responses to biotic stresses[1,2]. However, JAs modulate many developmental traits and, more recently, JAs were also involved in plant responses to abiotic stresses[2–4].

JAs regulate many physiological responses via a well-studied signalling pathway. Upon stress perception, plants generate JAs via the oxilipin biosynthetic pathway leading to the final accumulation of the bioactive JA-Ile molecule, the (+)-7-*iso*-JA-Ile[2,5]. JA-Ile induces the interaction between the F-box COI1 (CORONATINE INSENSITIVE 1)[6] and the JAZ co-receptors (JAMONATE-ZIM PROTEIN)[7,8] leading to the ubiquitination and subsequent degradation of the JAZ proteins[9]. The JAZ proteins act as repressors of several transcription factors (TFs); therefore the degradation of JAZ repressors liberates several TFs that in turn induce the cellular transcriptional reprogramming[10,11].

The JAZ proteins belong to the TIFY super-family, that also includes TIFY8, PPD and ZIM proteins[10–12]. The JAZ family of repressors is composed of 12 canonical members in Arabidopsis and an atypical repressor, JAZ13[10,13]. The JAZs are disordered proteins and contain two well-conserved domains regulating the interaction with several proteins and defining their activity[14,15]. The TIFY or ZIM domain, conserved in all TIFY proteins, induces the dimerization among JAZs and recruits the general transcriptional repressor TOPLESS (TPL) complex via the specific adaptor Novel Interactor of JAZ (NINJA)[16–18]. The Ethylene-responsive element binding factor-associated amphiphilic repression (EAR) domain of NINJA directly interacts and recruits TPL; different types of TPL-interacting EAR domains have been identified, being LxLxL or DLNxxP the predominant forms[19].

In contrast, the Jas motif is specific of the JAZ family and mediates the hormone-dependent interaction of the JAZ with the F-box COI1; besides, the JAZ repressors interact via the Jas motif with most TFs[9,10]. In addition, an N-terminal cryptic MYC2-interacting domain (CMID), conserved only in JAZ1 and JAZ10, also promotes the JAZ-TF interaction [20–22].

The Arabidopsis JAZ7 and JAZ8 retain a divergent Jas motif unable to interact with COI1; as a consequence, JAZ8 is not degraded by the perception of the hormone[23]. However, AtJAZ7 and AtJAZ8 retain a conserved EAR domain (LxLxL) able to directly recruit the general repressor TPL complex, bypassing the requirement of the adaptor NINJA protein [23–25]. Besides, the Jas-like motif of AtJAZ7 and AtJAZ8 mediates the interaction with several TFs involved in JA-dependent responses[23,25]. These data define a novel mechanism in JA signalling: the direct EAR-mediated recruitment of the general repressor TPL complex to the TFs regulating JA outputs [23]. In addition, AtJAZ5 and AtJAZ6 also retain two EAR motifs each, a C-term LxLxL and a DLNxxP adjacent to the ZIM domain[19]. Similarly, the atypical non-TIFY Arabidopsis JAZ13 interacts with different TFs regulating JA outputs via a divergent Jas-like motif and with TPL by a LxLxL EAR domain[13]. Finally, the AtJAZ13 lacks the ZIM domain and it is therefore unable to interact with other JAZ and with NINJA.

All TIFY proteins (TIFY8, PPDs and ZIMs) contain a ZIM/TIFY domain but lack the Jas motif, conserved only by JAZ proteins. TIFY8 acts as transcriptional repressor in complex with NINJA and TOPLESS[26]. In addition, PPD proteins are transcriptional regulators negatively regulating meristemoid division[27]. In addition, the leguminous ortholog *PPD* gene *BIG SEEDS1* regulates the plant organ size modulating primary cell proliferation[28]. ZIM and the closely related ZML1 and ZML2 proteins are GATA transcription factors involved in photoprotective response[29]. Recently, maize ZML2 was described as a transcriptional repressor of lignin biosynthesis [30]. The function of the ZIM domain, promoting ZIM dimerization and interaction with NINJA, is well conserved in all studied TIFY proteins[16,27,29].

JAZ repressors have been characterized in several economically important plant species such as wine grape, soya and rice among others[31–34]. In addition, many *TIFY* genes are significantly up-regulated by multiple abiotic stresses in different plant species and the over-expression of these stress-inducible *TIFY* gene increases tolerance to salt and drought in rice plants[32,35].

The discrete spatiotemporal expression of specific components of the ABA- and JA-pathway regulates the formation of different protein complexes that, in combination with differential affinity of protein-protein interactions, confers specificity to the hormone-mediated responses in specific tissues (e.g. roots compare to leaves)[10,36].

In tomato, an initial SlJAZ classification was reported as well as the transcriptional characterization of *SlJAZ* genes in response to the microbe *Pseudomonas syringae* and the bacterial phytotoxin COR, analog of the JA-Ile hormone[37,38]. In addition, the *SlJAZ*-silenced tomato plants showed enhanced cell death following *P*. *syringae* infection as well as delayed cell death in response to pathogen-associated molecular pattern, suggesting a primary role of tomato JAZs in cell death associated to bacterial infections[37]. However, an extensive characterization of the tomato *TIFY* gene family, including ZMLs, TIFY8 and PPDs, has not been reported so far. In addition, the transcriptional regulation of *SlTIFY* genes in response to abiotic stresses is very poorly explored[39–42].

Here, we describe a complete analysis of the *SlTIFY* family, including the newly identified *SlTIFY8*, *SlPPDs* and *SlZMLs*, and phylogenetic analyses defining the specific clades of the SlTIFY family. Finally, the transcriptional profile of *SlJAZ* genes in response to JA, ABA and abiotic stresses is reported. The importance of specific tissue expression in plant adaptation to abiotic stresses led us to evaluate gene expression in both root and above-ground tissues. Most *SlJAZ* are JA-induced and responsive to one or more abiotic stresses.

## Material and methods

### Sequence and phylogenetic analyses

In order to identify all members of the tomato *TIFY* gene family, BLAST searches with default parameters were employed using as prey the amino acid sequence of the complete Arabidopsis TIFY proteins, as well as of the TIFY and Jas motifs, in several Genome Databases: Plant Genome and Systems Biology (PGSB, www.pgsb.helmholtz-muenchen.de/plant/tomato), Sol Genomics Network (SGN, www.solgenomics.net) and Plants Ensembel (www.plants.ensembl.org).

The phylogenetic analysis was performed aligning the full-length protein sequences with DiAling (www.genomatix.de) and drawing the results trees with Tree Viewer tools (www.phylogeny.fr/). The MUSCLE program was employed for sequence alignment (www.ebi.ac.uk/Tools/msa/muscle/) and BoxShade for highlighting conserved residues.

The genomic data were obtained in Plants Ensembel (plants.ensembl.org) and the exon/intron structures drawn with Gene Structure Display Service (GSDS, www.gsds.cbi.pku.edu.cn/). The sequence logo for the TIFY domain and Jas motif were generated with Domain Drawer (www.weblogo.berkeley.edu).

The promoter sequences of all *SlJAZ* genes were obtained in Tomato Genomic Resources Database (www.59.163.192.91/tomato2/index.html) and analyzed with PlantCare (www.bioinformatic.psb.ugent.be/webtools/plantcare) [43].

### Plant growth conditions and hydroponic cultures

Seeds of *Lycopersicon esculentum* Mill. cultivar Micro-Tom were treated with nitric acid solution (0.05% v/v) for 1 min then washed 3 times with sterile water. Finally they were surface sterilized in 2.5% (v/v) sodium hypochlorite solution for 10 min followed by three rinses in sterile water. Seeds were sown in Petri dishes (10 per plates) containing solid (7g/l agar) MS (Murashige and Skoog, 1962) basal medium with 1% sucrose and germinated at 24°C during a 16 h light period and 8 h dark period. After 4–5 days of germination seedling with full-grown cotyledons were transferred to glass jars and grown in the same conditions until the development of the first true leaf. At this stage the plants were grown hydroponically in plastic boxes containing 1 l of ¼ x MS basal medium without vitamins for one month. These plants were treated with NaCl (150 mM), mannitol (300 mM), ABA (100 μM) and jasmonic acid (100 μM) during different times 0, 12, 24, 48 and 72 hours before collecting leaves and roots for total RNA extraction. Three plants for each time point were used as independent biological replicas. Then three technical replicas were employed for each transcriptional analysis.

### RNA extraction and genes expression profiling

Total RNA was isolated with the RNeasy Plant Mini Kit (QIAGEN) from 200 mg of leaf or root materials according to the manufacturer’s recommendations. The residual amount of DNA remaining in RNA was removed using on-column DNase I (PROMEGA) treatment during the RNeasy procedure. First strand cDNA synthesis was performed on 5 μg RNA using QuantiTect Reverse Transcription Kit (QIAGEN) with oligo (dT) 18 and random hexamer primers according to the manufacturer’s instructions. The reverse transcriptase (RT) reactions were diluted 1/3th and used as a template in real-time qPCR reactions. Primer pairs (FW and RV) were designed with Primer 3 software to amplify fragments of *SlJAZ1* to *SlJAZ12* genes and housekeeping *SlTublin* gene (S1 Table). For qPCR the amplification reactions were performed in 15 μl final volumes containing 7.5 μl of 2x concentrated LightCycler 480 SYBR Green I Master (ROCHE), 1.5 μl of primer-pair mix (0.5/0.5 lM for forward and reverse primers), 3 μl of cDNA and 3 μl of RNase-free water. Reactions were carried out in Light-Cycler 480 (ROCHE, Basel, Switzerland). The following reaction conditions were applied: 5 min at 95°C, followed by 40 cycles of 30 s at 95°C, 30 s at 60°C and 1 min at 72°C. At the end of the reaction the thermal cycler is programmed to produce the melting curve after the amplification cycles are completed as follow: plates read after one cycle 95°C for 10s followed by decreasing temperature to 60°C with a ramp of 4.4°C/s and finally by increasing temperature to 95°C with a ramp of 2.2°C every 1 s.

Each sample reaction was set up in triplicate to ensure the reproducibility of the results. cDNAs to be amplified (target and reference) were made with the same PCR master mix. The primer specificity was judged by melting-curve analysis and agarose gel electrophoresis of the amplification product. Furthermore, the products were sequenced (Macrogen, Korea) after inserted into pGEM-T easy vector (Promega) to confirm the primer pairs can specifically detect target genes by RT-qPCR. Each primer sequence was checked in the MiBase, KafTom and Solgenomics databases using a local BLAST search to ensure detect single gene. The primer pairs which matched with multiple uni-genes was excluded.

At the end of the reaction, the threshold cycle (CT) values of the triplicate PCRs were averaged and used for transcripts’ quantification. The relative expression was quantified by using the comparative CT method with the tomato tubilin (*SlTUB*) gene as an internal expression standard. The relative expression level was calculated as follows: 2-ΔΔCT, where ΔΔCT = (CTTarget gene—CTSlTubilin) stressed—(CTTarget gene—CTSlTubilin) control. The relative expression ratios from three independent technical replicates (composed of three independent biological samples) are reported. The fold-change in relative expression levels of the *SlJAZ* genes was transformed in log10-values and used for creating the pseudo-color heatmaps with HeatMapper Plus (BAR, www.bar.utoronto.ca/ntools/cgi-bin/ntools_heatmapper_plus.cgi).

## Results

### Identification and phylogenetic analysis of tomato *SlTIFY* genes

**Fig 1:**
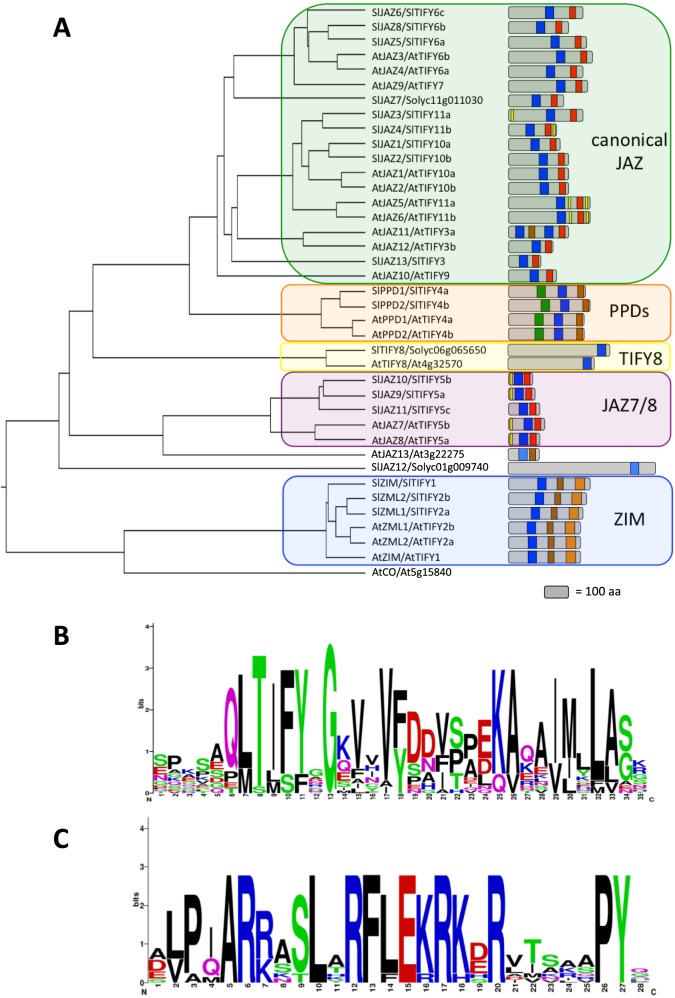
Sequence analysis of TIFYs in S. lycopersicum. (A) Phylogenetic analysis of *S*. *lycopersicum* and *A*. *thaliana* TIFY proteins. The phylogenetic tree was created in DiALing and TreeDraw softwares with the neighbor-joining method based on 20 full-length amino acids of SlTIFY proteins. The distribution of conserved domains in *S*. *lycopersicum* TIFY proteins. Each domain was represented by a colored box numbered at the bottom. The grey sections represented the non conserved sequences. The length of protein could be estimated using the grey box at the bottom (equals to 100 aa). Sequence logos of the TIFY (B) and Jas (C) domains from *S*. *lycopersicum* TIFY proteins.

To identify all putative tomato SlTIFY proteins, the Arabidopsis TIFY protein sequences were employed for search in different Genome Databases (PGSB, SGN and Plants Ensembel). We identified 20 non-redundant tomato loci encoding putative TIFY proteins (Table 1 and Fig 1), named according to previous reports[37,38]. All predicted tomato TIFY proteins contain the conserved TIFY domain (Fig 1 and S1 Fig), but only 9 SlTIFY show a conventional Jas motif among which the previously unreported canonical JAZ Solyc01g103600, named SlTIFY3/SlJAZ13 (Fig 1 and S2 Fig). Three additional SlTIFY exhibit a Jas-like motif similar to that of the divergent Jas of AtTIFY5 clade[23] (that includes AtJAZ7 and AtJAZ8; S3 Fig).

**Table 1:** List of *TIFY* genes from *Solanum lycopersicum*.

Gene name	Locus ID	Synonym	Protein length	TIFY motif	Jas motif	Duplications
SlTIFY1	Solyc01g106040	SlZIM	378	TISFEG	not present	Tandem
SlTIFY2a	Solyc10g047640	SlZML1	326	TLSFRG	not present	Segmental
SlTIFY2b	Solyc01g106030	SlZML2	311	TLSFQG	not present	Tandem
SlTIFY3	Solyc01g103600	SlJAZ13	129	TIFYAG	caninical	Dispersed
SlTIFY4a	Solyc06g084120	SlPPD1	319	TIFYRG	degenerate	Dispersed
SlTIFY4b	Solyc09g065630	SlPPD2	339	TIFYCG	degenerate	Dispersed
SlTIFY5a	Solyc08g036640	SlJAZ9	112	TIFYHG	divergent	Tandem
SlTIFY5b	Solyc08g036620	SlJAZ10	103	TIFYNG	divergent	Tandem
SlTIFY5c	Solyc08g036660	SlJAZ11	127	TIFYNG	divergent	Tandem
SlTIFY6a	Solyc03g118540	SlJAZ5	326	TIFYGG	caninical	Dispersed
SlTIFY6b	Solyc06g068930	SlJAZ8	246	TMFYAG	caninical	Dispersed
SlTIFY6c	Solyc01g005440	SlJAZ6	309	TIFYGG	caninical	Dispersed
SlTIFY7	Solyc11g011030	SlJAZ7	228	TIFYMG	caninical	Dispersed
SlTIFY8	Solyc06g065650	SlTIFY8	427	TIFYGG	not present	Dispersed
SlTIFY10a	Solyc07g042170	SlJAZ1	216	TIFYGG	caninical	Dispersed
SlTIFY10b	Solyc12g009220	SlJAZ2	252	TIFYGG	caninical	Dispersed
SlTIFY11a	Solyc03g122190	SlJAZ3	309	TMFYDG	caninical	Dispersed
SlTIFY11b	Solyc12g049400	SlJAZ4	200	SIFYGG	caninical	Dispersed
SlTIFY12	Solyc01g009740	SlJAZ12	636	TIFYDG	degenerate	Dispersed

**Fig 2:**
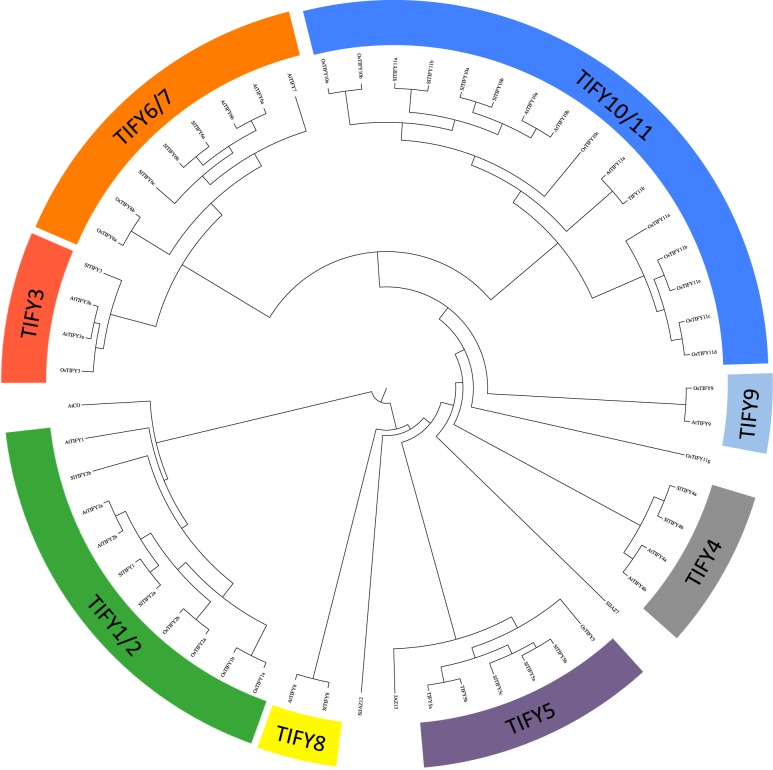
Phylogenetic tree of the TIFY family in plants. The phylogenetic tree shows the evolutionary relationships between proteins in the TIFY subfamily. Bootstrap values (>50%) for this tree are shown on respective branches. Members of different TIFY clades are highlighted in different colors.

In summary, we identified 20 SlTIFY including 12 SlJAZ (9 canonical and 3 divergent TIFY-like), 3 ZMLs, 2 PPDs, 1 TIFY8 and 1 putative atypical TIFY protein (Table 1 and Fig 1). The sequences of these 20 SlTIFY proteins were aligned with those of Arabidopsis and rice to generate a neighbour-joining phylogenetic tree (Fig 2 and S4 Fig). The proteins group into 8 major clades, corresponding to the previously reported TIFY classes[11,44]. In addition to JAZ proteins, three SlZIM and two SlPPD proteins were defined as well as a homolog of the AtTIFY8. The length of SlTIFY proteins varied extensively. As expected, the SlTIFY proteins belonging to the same group tend to contain similar protein length (Fig 1 and Table 1). In contrast, *SlJAZ12/Sl01g009740*, retaining a C-terminal divergent ZIM domain but lacking the Jas motif, encodes for an unusually long protein.

### Analysis of the ZIM domain and Jas motif

Next, we addressed the conservation of the domains of the tomato TIFY proteins. Among all SlTIFY proteins, the members of the TIFY clade 3, 6/7 and 10/11 are canonical JAZs with conventional ZIM domain and Jas motif (Figs 1 and 2). The sequence logo for the TIFY domain, generated using all SlTIFY, shows that the tomato TIFY domain is well conserved and very similar to those other plant species[44]. Besides, the Jas motif of canonical SlJAZ is strikingly similar to the consensus Jas motif SLX_2_FX_2_KRX_2_RX_5_PY (Fig 1B and S2 Fig). Noteworthy, the functional residues directly involved in the interaction between JAZ-COI1 and JAZ-hormone are remarkably conserved[45] (Fig 1C and S2 Fig). In addition, the residues essential for the interaction with MYC are also greatly conserved[46,47] (Fig 1C and S2 Fig). In addition, we searched for the cryptic MYC2-interacting domains (CMID) conserved in AtJAZ1 and AtJAZ10[17,21]. SlJAZ1/SlTIFY10a and SlJAZ2/SlTIFY10b show an N-terminal sequence very similar to the AtJAZ1/AtTIFY10a CMID (S5A Fig).

SlJAZ9, SlJAZ10 and SlJAZ11, showing a divergent Jas motif, belong to the TIFY5 clade that includes AtJAZ7 and AtJAZ8 (Fig 2 and S3 Fig). As the case of Arabidopsis, only two SlTIFY proteins exhibit the typical PPD domain, distinctive of the clade TIFY4[11,27]. Three SlTIFY proteins retain the conserved GATA domain characteristic of the TIFY1/2 clade (Figs 1 and 2). These analyses also define Solyc06g065650 as the tomato protein SlTIFY8, containing a C-terminal conserved TIFY domain but lacking the Jas motif. Finally, Sl01g009740/SlJAZ12, retaining only a divergent ZIM domain, fails to fit within any specific TIFY clade (Figs 1 and 2).

### Conservation of the EAR domain

The AtJAZ proteins belonging to the TIFY5 and TIFY11 clades (showing divergent and canonical Jas motif, respectively) preserve an EAR motif critical for recruiting the general repressor TPL machinery[18,23]. Two SlTIFY5 proteins retain a canonical LxLxL type EAR motif in the N-terminal region similarly to the Arabidopsis TIFY5[19] (S5B and S5C Fig). In contrast, the SlTIFY5c show a divergent N-terminal EAR domain (S5B and S5C Fig). In addition, SlTIFY7 (retaining a canonical Jas motif) shows a canonical NDLxxP type EAR motif, similarly to the second EAR motif of Arabidopsis TIFY5 proteins. Finally, in analogy with a second EAR NDLxxP type motif of Arabidopsis TIFY11 proteins, the two SlTIFY11 proteins retain an EAR-like motif of the same type[19] (S5B and S5C Fig).

### The genomic exon/intron structure of *TIFY* genes

The genomic exon/intron structure is an important indicator of the evolution of a gene family[32,48]. To further analyse the *SlTIFY* clades, the exon/intron structure of all *TIFY* genes of tomato and Arabidopsis was compared (S6 Fig). Overall, the exon/intron structure of *TIFY3*, *TIFY5*, *TIFY6/7* and *TIFY10/11* is similar among tomato and Arabidopsis genes. In contrast, the intron length of tomato *TIFY1/2* and *TIFY4* genes are longer than the Arabidopsis homologs, although the overall number and size of the exons are similar. Finally, *Sl01g009740/SlJAZ12* stands out because of its 23 exons compare with the average 5.8 exons in tomato *TIFY* genes (S6 Fig).

### *In silico* regulatory element analysis of *SlJAZ* gene promoters

Multiple biotic and abiotic stresses regulate the expression of several *JAZ* genes in different plant species [2,4]. Therefore, a 1.5 kb promoter region of the each *SlTIFY* gene was analysed using the PlantCARE database to identify putative stress-responsive *cis*-elements in the promoter regions of the tomato *TIFY* genes[43] (S2 Table). Several stress-responsive elements related to abiotic stresses were detected, including drought-inducible elements (MBS; T/CAACTG) and ABA-responsive elements (ABRE; ACGTGG/TC). In addition, specific *cis*-elements involved in JA-mediated responses, such as the MYC-binding G-box (CACGTG) and the MeJA-responsive elements (TGACG and CGTCA), were also identified (S2 Table). Overall, these data suggest that tomato *JAZ* genes are likely regulated by hormones and abiotic stresses.

### Expression profiles of *SlJAZ* genes in response JA and ABA

**Fig 3:**
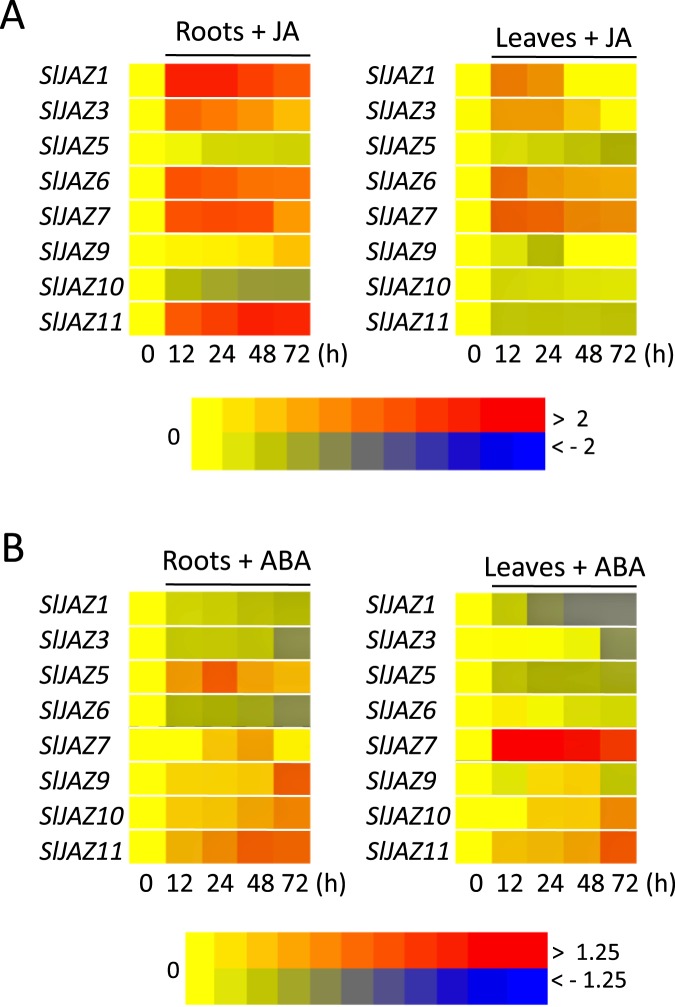
Expression profiles of *SlTIFY* genes in response to jasmonic and abscisic acid. Three-week-old tomato seedlings were subjected to exogenous JA (B) or ABA (B) treatments for different times; roots and leaves were collected and analyzed separately. Relative expression levels of the *SlTIFY* genes were analyzed by real-time quantitative RT-PCR (qPCR), and log10-transformed fold-change values were used for creating the heatmap (original data were shown in S3 Table). The colour-based gene expression scale is shown at the bottom of each panel.

To assess experimentally the transcriptional regulation of the *JAZ* genes in tomato plants, we analysed the long-term expression of several tomato *JAZ* genes in response to plant hormones JA and ABA. We assessed the transcriptional regulation in root and aboveground tissues to address the tissue-specific expression. First, we employed specific primers for all tomato *JAZ* genes of the tomato cultivar Micro-Tom (S1 Table) and confirm specific amplification for eight *SlJAZ* gens by sequence analysis. In agreement with the current knowledge, the heat map representation of transcript expression fold change shows that most *SlJAZ* genes are responsive to JA (Fig 3A and S3 Table). The *SlJAZ* induced by JA treatment in roots, are also triggered in the leaves with the exception of *SlJAZ11*, showing a root-specific JA-induction. None of the *SLJAZ* is strongly repressed by JA treatment. These data resemble the early *SlJAZ* expression reported in response to COR, the bacterial functional analog of JA-Ile[37]. These data are also consistent with the *in silico* analysis (S2 Table) since the *SlJAZ* genes with most conserved JA-responsive elements, such as *SlJAZ1* and *SlJAZ3*, are strongly induced by JA treatment.

ABA also induces the expression of several *SlJAZ* genes, although to a lesser extend than JA (Fig 3B). Five *JAZ* genes are consistently induced by ABA in tomato roots, but only *SlJAZ7* and *SlJAZ11* are also robustly up-regulated in leaves. In addition, ABA progressively represses the expression of *SlJAZ1* and *SlJAZ3* in both roots and leaves (Fig 3B).

In summary, both JA and ABA regulate the expression of several *SlJAZ* genes; some of them respond in an analogous fashion (e.i. *SlJAZ7* and *SlJAZ11* up-regulated by both JA and ABA), whereas others are differentially expressed (e.i. *SlJAZ1*, *SlJAZ3* and *SlJAZ36* up-regulated by JA but down-regulated by ABA).

### Expression profiles of *SLJAZ* genes in response to salinity and osmotic stresses

**Fig 4:**
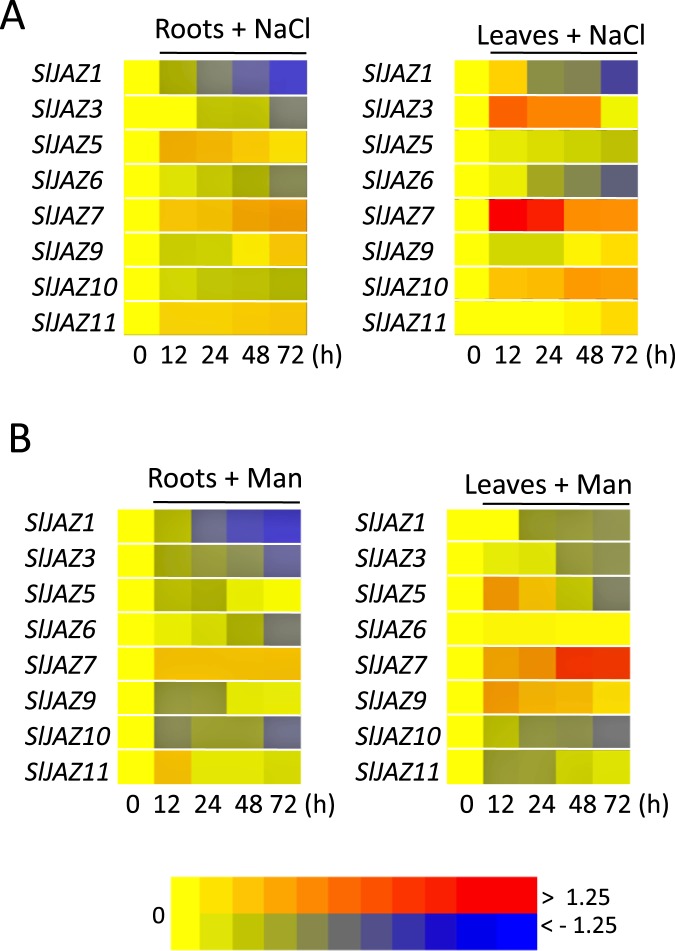
Expression profiles of *SlTIFY* genes under abiotic stress conditions. Expression profile of *SlTIFY* genes in response to salinity (A) orosmotic stresses (B). Three-week-old tomato plants were subjected to salt or osmotic stresses; roots and leaves were collected at different times and analyzed separately. Relative expression levels of the *SlTIFY* genes were analyzed by real-time quantitative RT-PCR (qPCR), and log10-transformed fold-change values were used for creating the heatmap (original data in S3 Table). The colour-based gene expression scale is included.

We also assess the effect of salinity and osmotic stress on *SlJAZ* expression (Fig 4A). Three *JAZ* (*SlJAZ3*, *SlJAZ7* and *SlJAZ10*) are consistently induced by salt in tomato leaves, but only *SlJAZ7* is also up-regulated in roots. In contrast, salt gradually represses the transcription of *SlJAZ1* and, to a minor extend, of *SlJAZ6* in both roots and leaves (Fig 4A and S3 Table). Thus, salt regulation of *SlJAZ* expression resembles that in response to ABA treatment.

Finally, six of the eight *SlJAZ* genes tested are down-regulated by osmotic stress in tomato roots (Fig 4B). In addition, transcription of *SlJAZ1*, *SlJAZ3* and *SlJAZ10* is also repressed in leaves. In contrast, *SlJAZ7* is the only gene up-regulated by osmotic stress.

The analysis of *SlJAZ* gene expression regulation in different tissues shows a comparable expression pattern since most *SlJAZ* expressions are similarly regulated in stressed root and distal leaves. Exceptionally, the robust induction in roots of *SlJAZ11* by JA and *SlJAZ5* by ABA or salinity stress respectively is not conserved in leaves (Fig 3). In an opposite fashion, *SlJAZ5* is repressed in roots by salinity stress but induced in distal leaves (Fig 4).

In summary, salinity regulates the expression of several *SlJAZ* genes both positively and negatively, whereas osmotic stress mainly represses *SlJAZ* genes.

## Discussion

Plant adaptation to changing environments relies on signalling networks. JAs play a central role in plant adaptation since they mediate several biotic responses and recently an important role of JAs in tolerance to abiotic stresses is emerging[2,4]. The JAZ proteins, belonging to the TIFY plant-specific family, are key components of the JA-mediated stress responses[10]. Previous studies described the tomato *JAZ* genes and their role in biotic interaction, but the comprehensive analysis of the tomato TIFY family has not been carried out yet[37,38]. Our analysis of the tomato TIFY family defines 7 novel members, including a novel canonical *JAZ* gene (Solyc01g103600) named *SlTIFY7/SlJAZ13*. Overall, tomato plants encode for 20 TIFY members, consisting of 12 SlJAZ (9 canonical and 3 divergent JAZs), 3 ZMLs, 2 PPDs, 1 TIFY8 and 1 putative atypical TIFY protein (Fig 1 and Table 1). This is consistent with the presence of these four TIFY sub groups in dicots[12,32,44].

The conserved ZIM/TIFY domain, usually located in the middle portion of the protein, defines the TIFY proteins. In addition, the members of the JAZ family also retain the characterising Jas motif at the C-terminal section. All SlTIFY retain the TIFY domain located in the expected middle portion of the protein, with the exception of the previously annotated SlJAZ12/Sl01g009740, showing a C-terminal divergent ZIM domain (Fig 1). In addition, SlJAZ12/Sl01g009740 does not conserve the Jas motif, suggesting that SlJAZ12/Sl01g009740 may not belong to the JAZ. However, the SlJAZ12/Sl01g009740 retains a degenerated TIFY domain and might be considered a member of the TIFY family. Moreover, a sub-group of JAZ repressors, such as AtJAZ8, retain an EAR domain to recruit directly the general transcriptional repressor TPL. Four SlJAZ proteins also show canonical EAR domain, suggesting that these tomato proteins might attract directly the TPL-recruited repression machinery (S5 Fig).

The Jas motif defines the JAZ proteins within the TIFY family. The Jas motif mediates the direct interaction JAZ-MYC as well as the hormone-dependent JAZ-COI1 complex formation. The residues directly involved in the interaction between JAZ-COI1 (Leu/Val, Pro, Ala, Arg and Arg/Lys in position 2, 3, 5, 6 and 7 of Jas motif respectively)[45] and JAZ-hormone (Alain position 5 of Jas motif)[45] are conserved among all SlJAZ proteins, including the newly reported SlJAZ13/Sl01g103600 (Fig 1C and S2 Fig). Remarkably, all residues involved in the direct JAZ-MYC interaction (R223, S226, L227, R229, F230, L231 and R234 in position 6, 9, 10, 12, 13, 14 and 17 of Jas motif respectively)[47] are also conserved in all SlJAZ proteins, including the newly annotated SlJAZ13/Sl01g103600 (Fig 1C and S2 Fig). Overall, the conservation of the key functional residues of the Jas motif suggests that the described SlJAZ proteins may act as functional JAZ repressors in tomato.

In addition, the AtJAZ12 is the only Arabidopsis JAZ protein that interacts with the RING E3 ubiquitin ligase KEEP ON GOING (KEG), a key repressor of the ABA pathway[49]. ABA triggers the KEG-dependent degradation of AtJAZ12 but not other JAZ proteins, suggesting a unique function of JAZ12 in JA/ABA crosstalk. The lack of a clear tomato *AtJAZ12* ortholog suggests differences in JA-ABA crosstalk in tomato plants compare to Arabidopsis. Alternatively, other SlJAZ proteins might interact with SlKEG and be degraded by ABA. Future experimental analyses will address the post-transcriptional ABA regulation of SlJAZ proteins.

The analysis of exon/intron organization of the tomato *TIFY* family shows the tomato and Arabidopsis *TIFY* genes show a clear similarity in exon/intron structure, with the exception of the ZIM and TIFY8 clades (S6 Fig). In accordance to previous tomato-Arabidopsis comparative genomic analyses, the number of introns present in the *TIFY* genes of both species is very similar[50]. In addition, most SlJAZ genes show an in-frame stop codon in the Jas-intron, similarly to other plant species (S6 Fig)[20]. Alternative splicing involving retention of the Jas-intron generate truncated JAZ proteins acting as constitutive repressors of the JA-pathway, such as the case of AtJAZ10.4[17,20]. Transcriptomic analyses will assess the occurrence in nature of these putative alternative-splicing events in tomato and their putative biological role.

The importance of the JAZ protein in mediating JA-regulated responses is well described, as well as their transcriptional up-regulation in response to JA accumulation[4,7,8]. Consistently, JA also induced most of the analysed tomato *JAZ* genes (Fig 3A). In addition, the extensive transcriptional characterization of *TIFY* family genes in rice and *Brachypodium distachyon* highlighted that most *OSTIFY* and *BdTIFY* genes were significantly up-regulated by multiple abiotic stresses[32,33]. The over-expression of the stress-inducible *OsTIFY11* gene confers increased tolerance to salt and drought in rice plants, confirming the involvement of the JAZ proteins in abiotic stress responses [35]. ABA is the key hormone in plant responses to environmental and abiotic stresses. Several *JAZ* genes of different plant species are modulated by ABA treatment[4,32,33,35]. Here, we show that *SlJAZ7* and *SlJAZ11* genes are also induced by ABA, and these ABA-induced *SlJAZ* genes are up-regulated by at least one abiotic stress treatment (Figs 3B and 4). In addition, a role of stress-induced *OsJAZ9/OsTIFY11a* in plant responses to abiotic stresses was described in rice [4,33,35,51]. Over-expression and suppression of *OsJAZ9/OsTIFY11a* resulted in increased and reduced tolerance to salt and dehydration stresses respectively[35,51]. Our transcriptional analysis showed that *SlJAZ3/SlTIFY11a* retains stress-responsive *cis*-elements in its promoters and was induced by salinity stresses in tomato leaves (Fig 4). In contrast, the transcription of other tomato *JAZ* genes was negatively regulated by abiotic stresses, especially in roots, as reported for other plant species[32].

Genes induced by environmental stresses or phytohormones such as JA are often involved in mediating the adaptive responses in these stresses or phytohormones[52,53]. Drought and salinity stresses induce accumulation of ABA, JAs and consequently the expression of many ABA- and JA-regulated genes mediating downstream physiological responses[3,54]. For example, salt stress induced the biosynthesis of ABA and JA, which subsequently triggered the activation of JA-responsive gene expression and, ultimately, nicotine synthesis in tobacco plants[55]. The regulation of *SlJAZ5*, *SlJAZ7* and *SlJAZ11* genes by ABA and multiple environmental stresses suggest a putative role for these stress-induced *SlJAZ* genes in environmental stress responses (Figs 3 and 4).

Specific tissue regulation plays a primary role in mediating a diversity of physiological processes and adaptive responses to environmental stresses[56]. For example, ABA receptors and signalling components in tomato are differentially expressed in different tissues, preferentially in roots, and specific ABA-independent and ABA-dependent protein-protein interactions occur[36]. Similarly, in the JA signalling pathway, despite a large redundancy in the signalling components, specificity is mainly achieved by distinct protein–protein interactions forming unique JAZ/transcription factor complexes, and discrete spatiotemporal expression of specific components[10]. Here, we show that the expression of *SlJAZ* in roots compare to leaves in response to hormones and abiotic stresses show an overall similar expression pattern (Figs 3 and 4). However, the strong induction of specific *SlJAZ* in stressed roots but not in distal leaves (e.i. *SlJAZ11* by JA and *SlJAZ5* by ABA or salinity stress) suggest that these JAZ proteins may form specific complexes in different tissues to regulate exclusive stress responses (Fig 3). Such is the case of RSS3 (RICE SALT SENSITIVE3) that is preferentially expressed in root tip and forms a ternary complex with transcription factors and JAZ proteins in rice during adaptation to salinity[31].

In conclusion, 20 *bona fide TIFY* genes were identified in *S*. *lycopersicum*. Gene expression analyses showed that JA induced most *SlJAZ* genes. Several *SlJAZ* genes were also responsive to salinity, osmotic stress or ABA treatment. Overall, the transcriptional regulation of the *SlJAZ* genes suggests a role for SlJAZ in JA-mediated responses as well as in abiotic stress responses. These results open the way for further functional characterization of *JAZ* genes in abiotic stress responses in tomato.

## Supporting information

S1 Fig Multiple sequence alignment of the conserved TIFY domain of several TIFY proteins. The alignment of the conserved TIFY domain of all TIFY proteins analyzed in this report. Sequences of tomato and Arabidopsis TIFY proteins were employed. Gray-shaded and black-shaded residues indicate conservation (amino acid identity) in at least 50% (grey) or all (black) amino acids of the aligned proteins respectively. The MUSCLE program was employed for sequence alignment and BoxShade for highlighting conserved residues and generating the consensus sequence.
(PDF)

S2 Fig Multiple sequence alignment of the canonical Jas motif of several JAZ proteins. The alignment of the sequences of the conserved Jas motif of several JAZ proteins is shown. Sequences of canonical tomato and Arabidopsis JAZ proteins were employed. Gray-shaded and black-shaded residues indicate conservation (amino acid identity) in at least 50% (grey) or all (black) amino acids of aligned proteins respectively. The residues (highlighted in yellow) critical for JAZ interaction with the MYC transcription factors are very conserved (Withers *et al*. 2012; Zhang et al., 2015). The residue directly interacting with COI1 (highlighted in blue) and the hormone JA-Ile (Ala in position 5 highlighted in bold) are also very conserved (Sheard et al., 2010). The MUSCLE program was employed for sequence alignment and BoxShade for highlighting conserved residues and generating the consensus sequence.
(PDF)

S3 Fig Multiple sequence alignment of divergent Jas of several TIFY proteins. The alignment of the sequences of the divergent Jas domain of several proteins belonging to the TIFY5 clade is shown. Sequences of tomato, Arabidopsis and rice TIFY5 proteins were employed. Gray-shaded and black-shaded residues indicate conservation (amino acid identity) in at least 50% (grey) or all (black) amino acids of the six aligned proteins respectively. The residues (highlighted in yellow) critical for JAZ interaction with the MYC transcription factors are very conserved in all TIFY5 proteins (Withers *et al*. 2012; Zhang et al., 2015). The residue directly interacting with COI1 and the hormone JA-Ile are not present in the divergent Jas of TIFY5 proteins (Sheard et al., 2010). The MUSCLE program was employed for sequence alignment and BoxShade for highlighting conserved residues and generating the consensus sequence.
(PDF)

S4 Fig Multiple sequence alignment of the full-length TIFY proteins. The alignments of the complete sequences of the TIFY proteins are shown. Sequences of tomato, Arabidopsis and rice proteins were employed. Gray-shaded residues indicate conservation (amino acid identity) in at least 50% of the aligned proteins, whereas residues conserved in all proteins are highlighted in black. The MUSCLE program was employed for sequence alignment and BoxShade for highlighting conserved residues and generating the consensus sequence.
(PDF)

S5 Fig Multiple sequence alignment of the CMID and the EAR motif in several TIFY proteins. The alignments of the amino acidic sequences of the cryptic MYC2 interacting domain (CMID) (A), the EAR LxLxL type (A) and NDLxxP type (B) are shown. Sequences of tomato, Arabidopsis and rice CMID- and EAR-containing proteins were employed. SlTIFY5c, SlTIFY11a and SlTIFY11b show divergent EAR-like motifs. The three conserved leucine residues of the EAR LxLxL type are highlighted in orange (A), whereas the conserved residues of the EAR NDLxxP type (B) are highlighted in yellow. Gray- and black-shaded residues indicate conservation (amino acid identity) in at least 50% or all of the aligned proteins respectively. The MUSCLE program was employed for sequence alignment and BoxShade for highlighting conserved residues and generating the consensus sequence.
(PDF)

S6 Fig Exon/Intron structures of TIFY genes. Different exon/intron structures of TIFY (JAZ, PPD and ZML) genes in *S*. *lycopersicum* and *A*. *thaliana* are shown. Blue boxes represent exons and black lines stand for introns. UTR regions are represented as grey boxes. Red asterisks represent Jas-intron retaining an in-frame stop codon. The length of the gene (in kilo bases) is annotated on the right and the scale is included at the bottom.
(PDF)

S1 Table Specific primers for the JAZ genes of the tomato cultivar Micro-Tom. (PDF)

S2 Table PlantCARE *cis*-element analysis of the SlTIFY genes’ promoter regions. (PDF)

S3 Table Original real-time quantitative RT-PCR data. (PDF)
