# Supplementary material for: Identification of TIFY/JAZ family genes in Solanum lycopersicum and their regulation in response to abiotic stresses
Source: PLoS One. 2017 Jun 1;12(6):e0177381. doi: 10.1371/journal.pone.0177381 (PMC5453414; doi:10.1371/journal.pone.0177381)
Supplement: S1 Fig — The alignment of the conserved TIFY domain of all TIFY proteins analyzed in this report. Sequences of tomato and Arabidopsis TIFY proteins were employed. Gray-shaded and black-shaded residues indicate conservation (amino acid identity) in at least 50% (grey) or all (black) amino acids of the aligned proteins respectively. The MUSCLE program was employed for sequence alignment and BoxShade for highlighting conserved residues and generating the consensus sequence. (PDF) [file pone.0177381.s001.pdf]

```
                                  -------- TIFY motif --------
SlJAZ12/Sl01g009740        GVKVEPLTIFYDGKIVVYD-VSIEKATNILKFVER
SlJAZ11/Sl08g036660        EQKSEPLTIFYNGKLVV-SHVTDLQAKAIIYLASR
SlJAZ9/Sl08g036640         NKQSQQLTIFYHGKFVV-SDATELQAKAIIYLASR
SlJAZ10/Sl08g036620        NEQSQQLTIFYNGK-FVASHVTQLQAKAIIYLASR
AtJAZ7/At2g34600           KQESQILTIFYNGHMCVSSDLTHLEANAILSLASR
AtJAZ8/At1g30135           NEESQRITIFYNGKMCFSSDVTHLQARSIISIASR
AtJAZ11/At3g43440          MILPSQLTIIFGGSFSVFDGIPAEKVQEILHIAAA
AtJAZ12/At5g20900          TVPPNQLTIFFGGSVTVFDGLPSEKVQEILRIAAK
SlJAZ7/Sl11g011030         SPNESTLTIFYMGEVHIFPGISPEKAELIIDLVSK
AtZIM/At4g24470            PEGANQLTISFRGQVYVFDAVGADKVDAVLSLLGG
SlZIM/Sl01g106040          SSRTSELTISFEGQVYVFPAVTPEKVQAVMLLLGG
AtZML2/At1g51600           SEQGDQLTLSFQGQVYVFDSVLPEKVQAVLLLLGG
AtZML1/At3g21175           IENGDQLTLSFQGQVYVFDRVSPEKVQAVLLLLGG
SlZML2/Sl01g106030         SGASDQLTLSFQGEVYVFDAVSPEKVQAVLLLLGG
SlZML1/Sl10g047640         GGSSDQLTLSFRGEVFVYDAVSPEKVQAVLLLLGG
AtJAZ10/At5g13220          VSGTVPMTIFYNGSVSVFQ-VSRNKAGEIMKVANE
AtJAZ5/At1g17380           QPGSSQLTIFFGGKVLVYNEFPVDKAKEIMEVAKQ
AtJAZ6/At1g72450           ESGNSQLTIFFGGKVMVFNEFPEDKAKEIMEVAKE
AtJAZ1/At1g19180           ESQTAPLTIFYAGQVIVFNDFSAEKAKEVINLASK
AtJAZ2/At1g74950           ESQSAPLTIFYGGRVMVFDDFSAEKAKEVIDLANK
SlJAZ3/Sl03g122190         EPKAAQLTMFYDGKVIVFDDFPADKARAVMLLASK
SlJAZ4/Sl12g049400         EQKLAQLSIFYGGKVVVFDDFPAEKARAVMLLASK
SlJAZ1/Sl07g042170         EPEKAQMTIFYGGQVIVFNDFPADKAKEIMLMAST
SlJAZ2/Sl12g009220         QPEKAQMTIFYGGQVIVFDDFPADKANEIMKLANK
SlTIFY8/Sl06g065650        SPAGSQMTIFYGGQAHVFDNVHPNKADVIMSLAGS
AtTIFY8/At4g32570          ASSTKQMTIFYGGQAHVFDDVHPNKADVIMALAGS
SlJAZ13/Sl01g103600        TSESEQLTIFYAGIVHVYDNISVQKAESIMNLASE
SlPPD1/Sl06g084120         DKPVGQMTIFYRGKVNVYDDVPADKAQKIMCLASS
SlPPD2/Sl09g065630         NASSGQLTIFYCGKVNVYDDVPAEKAEAIMHLAAS
AtPPD1/At4g14713           SELVGQMTIFYSGKVNVYDGIPPEKARSIMHFAAN
AtPPD2/At4g14720           NAVVGQMTIFYSGKVNVYDGVPPEKARSIMHFAAN
AtJAZ9/At1g70700           SGSSPQLTIFYGGTISVFNDISPDKAQAIMLCAGN
SlJAZ6/Sl01g005440         SAAPAQLTIFYGGMVNVFEDISPEKAQAIMFLAGH
AtJAZ4/At1g48500           KPLPPQLTIFYAGSVLVYQDIAPEKAQAIMLLAGN
AtJAZ3/At3g17860           IGSPAQLTIFYAGSVCVYDDISPEKAKAIMLLAGN
SlJAZ5/Sl03g118540         PPGPAQLTIFYGGSVCVYDNVSPEKAQAIMLLAGN
SlJAZ8/Sl06g068930         SPGPAQLTMFYAGSVCVYDNISPEKAQAIMLLAGN
consensus                       qltify G v vfd vs eka aim laa
                                 ::: : *    .       :.  ::
```
