# Supplementary material for: Identification of TIFY/JAZ family genes in Solanum lycopersicum and their regulation in response to abiotic stresses
Source: PLoS One. 2017 Jun 1;12(6):e0177381. doi: 10.1371/journal.pone.0177381 (PMC5453414; doi:10.1371/journal.pone.0177381)
Supplement: S2 Fig — The alignment of the sequences of the conserved Jas motif of several JAZ proteins is shown. Sequences of canonical tomato and Arabidopsis JAZ proteins were employed. Gray-shaded and black-shaded residues indicate conservation (amino acid identity) in at least 50% (grey) or all (black) amino acids of aligned proteins respectively. The residues (highlighted in yellow) critical for JAZ interaction with the MYC transcription factors are very conserved (Withers et al. 2012; Zhang et al., 2015). The residue directly interacting with COI1 (highlighted in blue) and the hormone JA-Ile (Ala in position 5 highlighted in bold) are also very conserved (Sheard et al., 2010). The MUSCLE program was employed for sequence alignment and BoxShade for highlighting conserved residues and generating the consensus sequence. (PDF) [file pone.0177381.s002.pdf]

```
                         ------ Jas motif ---------
SlJAZ7/Sl11g011030       ALAMARRATLARFLEKRKHRLIKARPYL
AtJAZ11/At3g43440        DVPIARRRSLQRFFEKRRHRFVHTKPYS
AtJAZ4/At1g48500         GLPQTRKASLARFLEKRKERVINVSPYY
AtJAZ3/At3g17860         ALPLARKASLARFLEKRKERVTSVSPYC
SlJAZ6/Sl01g005440       SVPQARKASLARFLEKRKERVMNLAPYG
AtJAZ9/At1g70700         SVPQARKASLARFLEKRKERLMSAMPYK
SlJAZ5/Sl03g118540       AVPQARKASLARFLEKRKERVISASPYP
SlJAZ8/Sl06g068930       AVPQARKASLARFLERRKERVVSASPYG
AtJAZ5/At1g17380         VERIARRASLHRFFAKRKDRAVARAPYQ
AtJAZ6/At1g72450         VERIARRASLHRFFAKRKDRAVARAPYQ
SlJAZ13/Sl01g103600      ELPIARRKSLKRFFEKRHSRITSKQPYA
SlJAZ1/Sl07g042170       DLPIARRASLTRFLEKRKDRLTAKVPYH
SlJAZ2/Sl12g009220       DLPIARRNSLTRFLEKRKDRVTSIAPYQ
AtJAZ1/At1g19180         ELPIARRASLHRFLEKRKDRVTSKAPYQ
AtJAZ2/At1g74950         ELPIARRASLHRFLEKRKDRITSKAPYQ
SlJAZ4/Sl12g049400       DLPIARRSSLYRFLEKRKDRDTARAPYQ
SlJAZ3/Sl03g122190       ELPIARRSSLHRFLEKRKDRATVRAPYQ
AtJAZ10/At5g13220        DLPIARRKSLQRFLEKRKERLVSTSPYY
AtJAZ12/At5g20900        DLPIARRHSLQRFLEKRRDRLVNKNPYP
consensus                dlpiaRrasL RFlekRkeRli   PY
                             :*. :* **: .*. *     **
```
