# Supplementary material for: Identification of TIFY/JAZ family genes in Solanum lycopersicum and their regulation in response to abiotic stresses
Source: PLoS One. 2017 Jun 1;12(6):e0177381. doi: 10.1371/journal.pone.0177381 (PMC5453414; doi:10.1371/journal.pone.0177381)
Supplement: S3 Fig — The alignment of the sequences of the divergent Jas domain of several proteins belonging to the TIFY5 clade is shown. Sequences of tomato, Arabidopsis and rice TIFY5 proteins were employed. Gray-shaded and black-shaded residues indicate conservation (amino acid identity) in at least 50% (grey) or all (black) amino acids of the six aligned proteins respectively. The residues (highlighted in yellow) critical for JAZ interaction with the MYC transcription factors are very conserved in all TIFY5 proteins (Withers et al. 2012; Zhang et al., 2015). The residue directly interacting with COI1 and the hormone JA-Ile are not present in the divergent Jas of TIFY5 proteins (Sheard et al., 2010). The MUSCLE program was employed for sequence alignment and BoxShade for highlighting conserved residues and generating the consensus sequence. (PDF) [file pone.0177381.s003.pdf]

```
                                    --------- Jas motif---------
SlTIFY5a/SlJAZ9/Sl08g036640   -SSPLLQPQTVKKSLQGFLQKRKKRVQATSPYHK--
SlTIFY5b/SlJAZ10/Sl08g036620  -SSPLLQPQTVKKSLQRFLQKRKNRIQITSPYHH--
SlTIFY5c/SlJAZ11/Sl08g036660  -SSPLLQPQTVKKSLQRFLQKRKSRTQTTSPYHH--
AtTIFY5a/AtJAZ8/At1g30135     HNQLPNPKASMKKSLQSFLQKRKIRIQATSPYHSRR
AtTIFY5b/AtJAZ7/At2g34600     STRFHYQKASMKRSLHSFLQKRSLRIQATSPYHRYR
OsTIFY5/Os07g05830            NQPPAASGLSMKRSLQRFLEKRKTRAAA-PLYA-RR
                                       ::*:**: **:**. *      *
```
