# Supplementary material for: Identification of TIFY/JAZ family genes in Solanum lycopersicum and their regulation in response to abiotic stresses
Source: PLoS One. 2017 Jun 1;12(6):e0177381. doi: 10.1371/journal.pone.0177381 (PMC5453414; doi:10.1371/journal.pone.0177381)
Supplement: S4 Fig — The alignments of the complete sequences of the TIFY proteins are shown. Sequences of tomato, Arabidopsis and rice proteins were employed. Gray-shaded residues indicate conservation (amino acid identity) in at least 50% of the aligned proteins, whereas residues conserved in all proteins are highlighted in black. The MUSCLE program was employed for sequence alignment and BoxShade for highlighting conserved residues and generating the consensus sequence. (PDF) [file pone.0177381.s004.pdf]

```
SlJAZ7/SlTIFY7        1 -----------------------MDSRMEIDFMDLNSK---------------------P--------------K-LSEMEKQH-----------------------------------K
AtJAZ11/AtTIFY3a      1 MAEVNGDFPVPSFADGTGSVSAGLDLLVERSIHEARST-EPDAST--------------Q----LTIIFGGSCRV-FNGVPAQK----------------------------------VQ
AtJAZ12/AtTIFY3b      1 --------------------------------MTKVKD-EP------------------------RASVEGGCGV-ADGDGG--------------------------------------
AtJAZ5/AtTIFY11a      1 --------------------------------MSSSNE-NAKAQA--------------P----EKSDFTRRCSL-LSRYLKEK-----------------------------------G
AtJAZ6/AtTIFY11b      1 ---------------------------------------MSTGQA--------------P----EKSNFSQRCSL-LSRYLKEK-----------------------------------G
SlJAZ3/SlTIFY11a      1 ----------------------------MSNLCDARRRN-GNGKA--------------P----ERSSFVQTCNL-LSQFIKGK-----------------------------------A
SlJAZ4/SlTIFY11b      1 ----------------------------------MSNRQ-LCSLD--------------S----EKSHLMNTCNL-LTQFFNGK-----------------------------------A
SlJAZ13/SlTIFY3       1 ------------------------------------------------------------------------------------------------------------------------
AtJAZ1/AtTIFY10a      1 ----------------------------MSSSMECSEFVGSRRFT--------------G----KKPSFSQTCSR-LSQYLKEN-----------------------------------G
AtJAZ2/AtTIFY10b      1 ---------------------------MSSFSAE------CWDFS--------------G----RKPSFSQTCTR-LSRYLKEK-----------------------------------G
SlJAZ1/SlTIFY10a      1 --------------------------------MASSEIVDSGRFA--------------G----QKSHFSHTCNL-LSQYLKEK----------------------------------KG
SlJAZ2/SlTIFY10b      1 --------------------------------MGSSENMDSGKVT--------------G----QKSQFSQTCNL-LSQFLKKK-----------------------------------G
AtJAZ10/AtTIFY9       1 ----------------------MSKATIELDFLGLEKKQTNNAPK--------------------------------PKFQKFLD--------------------------------RRR
SlJAZ11/SlTIFY5c      1 ------------------------------------------------------------------------------------M--------------------------------RRN
SlJAZ10/SlTIFY5b      1 ------------------------------------------------------------------------------------M--------------------------------RRK
SlJAZ9/SlTIFY5a       1 ------------------------------------------------------------------------------------M--------------------------------RRN
AtJAZ7/AtTIFY5b       1 -------------------------------MIII--IKNCDKPL--------------------------------LNFKEMEM--------------------------------QTK
AtJAZ8/AtTIFY5a       1 ----------------------------------------------------------------------------------MKL--------------------------------QQN
AtJAZ9/AtTIFY7        1 ---------------------------MERDFLGLSDKQYLSNNVKHEVNDDAVEERGLSTKAAREWGKSKVFAT--SSFM-------------------------P-------------
AtJAZ3/AtTIFY6b       1 ---------------------------MERDFLGLGSKNSPITVK-EETSESSRD--SAPNRG-MNWSFSNKVSASSSQFLSFRPTQEDRHRKSGNYHLPHSGSFMPSSVADVYDSTRKA
AtJAZ4/AtTIFY6a       1 ---------------------------MERDFLGLGSKLSPITVK-EETNED-----SAPSRGMMDWSFSSKVGS-GPQFLSFGTSQQETRVNTVNDHLLSSA---------AMDQNQRT
SlJAZ6/SlTIFY6c       1 ---------------------------MERDFMGLNIKDSLLVVK-DEPVESSKD--SG-----FRWPMSSKVGV--PHFMSLNSAQDE------------NT-----------------
SLJAZ8/SlTIFY6b       1 ----------------------------------------------------------------MHWSYSNKA---HPQYHLSLKDQENIIINNNNNNKPKIG-----------------
SlJAZ5/SlTIFY6a       1 ---------------------------MERDFMGLTVKQEVLEEP-IDPA-PLRS--SA-----MQWSFTNNVTA-HPQYL---------SFKSAPEDKPKIG-----------------
consensus             1                                 m                                   ft         yl

SlJAZ7/SlTIFY7       27 KVSGMKWPFSLADLAT--HH-EHTFFQNYK-----------STPIVSINSKN---SSLNNYKSTID---------PQYFRGTFPLLAK----TS--------TYDSRKNYDNLSPNESTL
AtJAZ11/AtTIFY3a     67 EI--IRIA--FAGKQT-----------------------KNVTGINPALNRA---LSF----STVA---------------DLPIARRRSLQRFLEK-----RRDRSTKPDGSMILPSQL
AtJAZ12/AtTIFY3b     25 ---------------------------------------------------------------------------------AAEIGGTGSVEKSINE-----VRSTEIQTAEPTVPPNQL
AtJAZ5/AtTIFY11a     34 SFGNIDLG--LYRKPDSSLA-LPGKFDPPGKQ-------N-------AMHK-------------AGHS-------KGEPS------------TSSGGKV---KDVA--DLSESQPGSSQL
AtJAZ6/AtTIFY11b     28 SFGNINMG--LARKSD--LE-LAGKFDLKGQQ-------N-------VIKK-------------VETS-------ETRPFKLIQKFSIGEASTSTEDKA-IYIDLSEPAKVAPESGNSQL
SlJAZ3/SlTIFY11a     38 TIRDLNLG--IAGQP----E-AAG-----KTE-------TATMDLLTVMEKP---SID------LTKE-------EHKSVDLVTTESSREKEAA------VNEPSTSKEA-PKEPKAAQL
SlJAZ4/SlTIFY11b     32 NINDLNLT--ISNNG----E-AKA---------------SATKDLLTNMEEL---STK------TTEQ-------DQKLIDHVPK-------SA------INKASGSKEIPHKEQKLAQL
SlJAZ13/SlTIFY3       1 --------------------------------------------------------------------------------MRIPRHDD----------------TKDPVSQRVTSESEQL
AtJAZ1/AtTIFY10a     39 SFGDLSLG--MACKP----D-VNGTLGNSRQP-------TTTMSLFPCEASN---MDS-----MV--Q-------DVKPTNLFPRQPSFSSSSSSLPKEDVLKMTQTTRSVKPESQTAPL
AtJAZ2/AtTIFY10b     34 SFGDLSLG--MTCKP----D-VNGGS---R-Q-------PTMMNLFPCEASG---MDS-----SAGQE-------DIKPKTMFPRQSSFSSSSSSGTKEDVQMIKETTKSVKPESQSAPL
SlJAZ1/SlTIFY10a     36 SLGDLSLD----MHRN--FD---------SAG-------STTMDLLPMIEKS---GELV-----------------QKSMNLFPQGGM---------------------KAESEPEKAQM
SlJAZ2/SlTIFY10b     35 SVGDLNNL--GIYKTT--FE-STGSQQTATTT-------TTTMNLLPMIEKS---SDSSSSSSSVETN-------PQKPMNLFPQEFDFSKEQSTKKT---------ESWKPDQPEKAQM
AtJAZ10/AtTIFY9      35 SFRDIQGAISKI---D--PEIIKSLLAST---------------GNNSDSSA--------KSRSVPSTPREDQP---------QIPIS------------PVHASLARSSTELVSGTVPM
SlJAZ11/SlTIFY5c      5 C--------------N--LE--FRLMP-P---------------SLSTF-SP--------NICSNNNTS------------------S------------YFSMEEDKESTELEQKSEPL
SlJAZ10/SlTIFY5b      5 C--------------N--LE--LTLSP-S---------------N-----------------LL---------------------------------------MEDK---RLENEQSQQL
SlJAZ9/SlTIFY5a       5 C--------------N--LE--LTLMP-P---------------SISDNFSS--------KNCT---------------------------------------TEDQ---QLENKQSQQL
AtJAZ7/AtTIFY5b      24 C--------------D--LE--LRLLT-S---------------SYDSDFHS----------SLDESSS--------------------------------------SEISQPKQESQIL
AtJAZ8/AtTIFY5a       7 C--------------D--LE--LRLFP-T---------------SYDSDSSD--------TTSVVESTS--------------------------------------SGNPQPNEESQRI
AtJAZ9/AtTIFY7       54 -SSDFQE---------------AKAFPGAYQWGSVSAANV--------FRRCQFGGAFQ----NATPLLLGGSV-------PLPTHPS------------LV------PRVASSGSSPQL
AtJAZ3/AtTIFY6b      90 PYSSVQG---------------VRMFPNSNQHEETN-AVSMSMPGFQSHHYA--PGGRSFMNNNNNSQPLVGVPIMAPPISILPPPGS------------IVGTTDIRSSSKPIGSPAQL
AtJAZ4/AtTIFY6a      78 YFSSLQE---------------DRVFPGSSQQDQTTITVSMSEP--------------NYINSFINHQHLGGSPIMAPPVSVFPAPTT------------I------RSSSKP--LPPQL
SlJAZ6/SlTIFY6c      55 -FKALSA---------------TD--------------------GVDAGLKR-QPGEL-----QMKQ-VLGGIP-VTAPHSMLPSRGS------------VAGTTEPWFNSKGSAAPAQL
SLJAZ8/SlTIFY6b      37 -FESLAS---------------AGLV-----------TITTTTELFDTIHRP-YTTQF--------------------GAHHVPTRNG------------VVGTTELRGTPRPSPGPAQL
SlJAZ5/SlTIFY6a      58 -FDSLAS---------------TGLV-----------TITTTE-AVDSSHRT-YSDVTQNLITTVNQLPGAGAL-VVSPISAVPSSSI------------VVGTTDLRGAPKTPPGPAQL
consensus           121     m              e                              k                                p                                  ql
```

```
SlJAZ7/SlTIFY7   109 TIFYMGEVHIFPGISPEKAELIIDLVSKSTTLHMDEILEKVMN---KEKY--E-------------------------------------------------------------------
AtJAZ11/AtTIFY3a 133 TIIFGGSFSVFDGIPAEKVQEILHIAAAAKATETINLTS-I---------------------------------------------------NPALKRAISFSN----------------
AtJAZ12/AtTIFY3b  59 TIFFGGSVTVFDGLPSEKVQEILRIAAKAMET--KNSTS-ISP---------VS--------------------------------------SPALNRAPSFSST---------------
AtJAZ5/AtTIFY11a 100 TIFFGGKVLVYNEFPVDKAKEIMEVAKQAKPVTEINIQTPINDENNNNKSSMVL---------------------------------------PDLN-EPTDN-----------------
AtJAZ6/AtTIFY11b 108 TIFFGGKVMVFNEFPEDKAKEIMEVAKEANHVAVDSKN--SQSHMNLDKSNVVI---------------------------------------PDLN-EPTSS-----------------
SlJAZ3/SlTIFY11a 116 TMFYDGKVIVFDDFPADKARAVMLLASKGCPQSSFGTFQAINI----DKINTCSPAPASLTSNRTDSVAPQQQHLQIKPDSCSAAPQQHKHNSPPLH-VCSSTKTDQLKLGS----VSS-
SlJAZ4/SlTIFY11b 101 SIFYGGKVVVFDDFPAEKARAVMLLASKGISNNSCAIFQTPTT----T------------------------------------------------------------------------
SlJAZ13/SlTIFY3   25 TIFYAGIVHVYDNISVQKAESIMNLASENCNAKE------IKP----TQK---------------------------------------------SQ-VP--------------------
AtJAZ1/AtTIFY10a 128 TIFYAGQVIVFNDFSAEKAKEVINLASKGTANSLAKNQTDIRS----------------------------------------------NIATIANQ-VPHP------------------
AtJAZ2/AtTIFY10b 121 TIFYGGRVMVFDDFSAEKAKEVIDLANKGSAKSFTCFTAEVNN----NHSAYSQ----------------------------------KEIASSPNP-VCSP------------------
SlJAZ1/SlTIFY10a  93 TIFYGGQVIVFNDFPADKAKEIMLMASTSKGNNP-AKP---------LES------------------------------------------AADLV-VPSFG-----------------
SlJAZ2/SlTIFY10b 124 TIFYGGQVIVFDDFPADKANEIMKLANKQNPTNNFTYPM--IK----NQK------------------------------------------TADQS-GVSFG-----------------
AtJAZ10/AtTIFY9  106 TIFYNGSVSVFQ-VSRNKAGEIMKVANEAASKKDESSME-----------------TDLSV------------------------------------------------------ILPT-
SlJAZ11/SlTIFY5c  52 TIFYNGKLVV-SHVTDLQAKAIIYLASRETEEKTNKSLS-----------------P-ISE--------------------------------------------------------P--
SlJAZ10/SlTIFY5b  32 TIFYNGKFVA-SHVTQLQAKAIIYLASREMEEKTNK----------------------LSE--------------------------------------------------------P--
SlJAZ9/SlTIFY5a   41 TIFYHGKFVV-SDATELQAKAIIYLASRGMEMKTNK----------------------MSE--------------------------------------------------------P--
AtJAZ7/AtTIFY5b   62 TIFYNGHMCVSSDLTHLEANAILSLASRDVEEKSLSLRS-----------------SDGSD--------------------------------------------------------P--
AtJAZ8/AtTIFY5a   47 TIFYNGKMCFSSDVTHLQARSIISIASREMKTKS----S-----------------SNGSD--------------------------------------------------------P--
AtJAZ9/AtTIFY7   121 TIFYGGTISVFNDISPDKAQAIMLCAGNGLKGETGDSKPVREAERMYGKQIHNTAATSSSSA----------------------------------------THTDNFSRCRDTPVAAT-
AtJAZ3/AtTIFY6b  180 TIFYAGSVCVYDDISPEKAKAIMLLAGNGSSMPQVFSPPQTHQQVVH----HTRASVDSSA--MPPSFMPTISY--LSPEAGSS--------------------TNGLGATKATRGLTST
AtJAZ4/AtTIFY6a  149 TIFYAGSVLVYQDIAPEKAQAIMLLAGNGPHAKPVSQP--KPQKLVH----HSLPTTDPPT--MPPSFLPSISY--IVSETRSS---------------GS-NGVTGLGPTKTKASLAST
SlJAZ6/SlTIFY6c  119 TIFYGGMVNVFEDISPEKAQAIMFLAGHGCAP-PNVVQPRFQLQASA----SKPAAADGVCVNQTPNMLPASGL--SSPMSVSS----HPI----GQSDGSSGNKDDMKMSKTANISVT-
SLJAZ8/SlTIFY6b   97 TMFYAGSVCVYDNISPEKAQAIMLLAGNTPISTTIRNSPSLDHHHH------------------------------------HH----H-N----NNNNNNNNSTNETTIIRSIGVLKS-
SlJAZ5/SlTIFY6a  136 TIFYGGSVCVYDNVSPEKAQAIMLLAGNAPPVTPNATSTLSPVQAPI----PKSSAIDSFVVNQCRNTTP--TL--ASPISITS----HGG----AQAARVSTTTNGVTIIKSIGVLPS-
consensus        241 tifygG vlvf dis ekakaim la ka

SlJAZ7/SlTIFY7   157 ----------ENKSD-PSN--ASTNYAKGALAMARRATLARFLEKRKHRLIKARPYLYGENLSKFPFDIQQ---------QEEETASSSVHWEN-------------------------------
AtJAZ11/AtTIFY3a 185 ------------------AS-TVACVSTADVPIARRRSLQRFFEKRRHRFVHTKPYSATTSEADKNETSPI--VT--------------------------------------------------
AtJAZ12/AtTIFY3b 114 -SNVAS---PAAQPF-PIQP-ISFCRSTADLPIARRHSLQRFLEKRRDRLVNKNPYPTSDFKKTDVPTGNV--SIKEEFPTA-------------------------------------------
AtJAZ5/AtTIFY11a 163 NHLTKE---QQ----------QQQEQNQIVERIARRASLHRFFAKRKDRAVARAPYQVNQNAGHHRYPPKPEIVTGQPLEAGQSSQRPPDNAIGQTMAHIKSDGDKDDIMKIEEGQSSKDLDLRL
AtJAZ6/AtTIFY11b 169 GNN--E---DQ----------ETGQQHQVVERIARRASLHRFFAKRKDRAVARAPYQVNQHGSH--LPPKPEMVAPS-IKSGQSSQHIATPPKPKAHNHM------PMEVDKKEGQSSKNLELKL
SlJAZ3/SlTIFY11a 226 APLVEQ---EQHKQI-QSQ--AAEISSSSELPIARRSSLHRFLEKRKDRATVRAPYQVVRNNPLLPSSSNT---------NGESSSKDSEDQLDLNFKL--------------------------
SlJAZ4/SlTIFY11b 145 ---------------------QTNGSNNFDLPIARRSSLYRFLEKRKDRDTARAPYQMHN--PLQ------------------SSSRTRGDHFDLNF----------------------------
SlJAZ13/SlTIFY3   69 ---------------------HHVYKFQAELPIARRKSLKRFFEKRHSRITSKQPYASPECDHDQS-----------------------ENWNDTKKINITH-----------AQE---------
AtJAZ1/AtTIFY10a 183 -----R---KTTTQE-PIQ--SSPTPL-TELPIARRASLHRFLEKRKDRVTSKAPYQLCDPAKASSNPQTT---------GN-------MSWLGLAAEI--------------------------
AtJAZ2/AtTIFY10a 184 -----A---KTAAQE-PIQ--PNPASLACELPIARRASLHRFLEKRKDRITSKAPYQIDGSAEASSKPTN-------------------PAWLSSR-----------------------------
SlJAZ1/SlTIFY10a 143 -----K---TSIQEN-Q----MPNQPIVSDLPIARRASLTRFLEKRKDRLTAKVPYHREEAAAPKK---EE---------HK-------APWLGLGGQFAVK-----------TEQY--------
SlJAZ2/SlTIFY10b 178 -----N---KLIQEL-PKL--SMPQPSVADLPIARRNSLTRFLEKRKDRVTSIAPYQISNNKKS-K---NE---------DN-------KAWLGLGAQFVKT-----------EQYF--------
AtJAZ10/AtTIFY9  153 ----------------TLRPKLFGQNLEGDLPIARRKSLQRFLEKRKERLVSTSPYYPTSA----------------------------------------------------------------
SlJAZ11/SlTIFY5c  95 -------------------------SSPLLQPQTVKKSLQRFLQKRKSRTQTTSPYHH-------------------------------------------------------------------
SlJAZ10/SlTIFY5b  71 -------------------------SSPLLQPQTVKKSLQRFLQKRKNRIQITSPYHH-------------------------------------------------------------------
SlJAZ9/SlTIFY5a   80 -------------------------SSPLLQPQTVKKSLQGFLQKRKKRVQATSPYHK-------------------------------------------------------------------
AtJAZ7/AtTIFY5b  107 ----------------PTIPNN--STRFHYQKASMKRSLHSFLQKRSLRIQATSPYHRYR-----------------------------------------------------------------
AtJAZ8/AtTIFY5a   88 ----------------PNKSTSFHHNQLPNPKASMKKSLQSFLQKRKIRIQATSPYHSRR-----------------------------------------------------------------
AtJAZ9/AtTIFY7   200 ---------NAMSMIESFNAAP--RNMIPSVPQARKASLARFLEKRKERLMSAMPYKKM---LLDLSTGESSGMNYS--------STSPT-----------------------------------
AtJAZ3/AtTIFY6b  272 YHNNQANGSNINCPV-PVSCSTNVMAPTVALPLARKASLARFLEKRKERVTSVSPYCLDKKSSTDCRRSMSECISSS--------LSSAT-----------------------------------
AtJAZ4/AtTIFY6a  243 RNN---------------QTAAFSMAPTVGLPQTRKASLARFLEKRKERVINVSPYYVDNKSSIDCRTLMSECVSCP--------PAHHLH----------------------------------
SlJAZ6/SlTIFY6c  223 PH-VKLDTSKIVTSLGPVGATTIMTAGMASVPQARKASLARFLEKRKERVMNLAPYGLSKK-SPECSTPESNGVGFS--------ATS----TPLLAGKET------------------------
SLJAZ8/SlTIFY6b  171 HE-LSKI---VTSQESRQPP----NHNLSAVPQARKASLARFLERRKERVVSASPYGNGKQSSQHMMNFT---------------INSSGSSTSLPAAN--------------------------
SlJAZ5/SlTIFY6a  239 PS-LKAEPSKVTSSVGSFPA----SLVPSAVPQARKASLARFLEKRKERVISASPYPLNSKQSPECSTPELGSRSLS--------MNSSGSCPPHIISLVK------------------------
consensus        361                               lpiarrasL rFlekRkdRl    PY
```
