# Supplementary material for: Identification of TIFY/JAZ family genes in Solanum lycopersicum and their regulation in response to abiotic stresses
Source: PLoS One. 2017 Jun 1;12(6):e0177381. doi: 10.1371/journal.pone.0177381 (PMC5453414; doi:10.1371/journal.pone.0177381)
Supplement: S5 Fig — The alignments of the amino acidic sequences of the cryptic MYC2 interacting domain (CMID) (A), the EAR LxLxL type (A) and NDLxxP type (B) are shown. Sequences of tomato, Arabidopsis and rice CMID- and EAR-containing proteins were employed. SlTIFY5c, SlTIFY11a and SlTIFY11b show divergent EAR-like motifs. The three conserved leucine residues of the EAR LxLxL type are highlighted in orange (A), whereas the conserved residues of the EAR NDLxxP type (B) are highlighted in yellow. Gray- and black-shaded residues indicate conservation (amino acid identity) in at least 50% or all of the aligned proteins respectively. The MUSCLE program was employed for sequence alignment and BoxShade for highlighting conserved residues and generating the consensus sequence. (PDF) [file pone.0177381.s005.pdf]

A

```
SlJAZ1/SlTIFY10a        MASSEIVDSGRFAGQKSHFSHTCNLLSQY
SlJAZ2/SlTIFY10b        MGSSENMDSGKVTGQKSQFSQTCNLLSQF
AtJAZ1/AtTIFY10a        MECSEFVGSRRFTGKKPSFSQTCSRLSQY
```

B

```
SlTIFY5a/SlJAZ9/Sl08g036640     CNLELTLM
SlTIFY5b/SlJAZ10/Sl08g036620    CNLELTLS
SlTIFY5c/SlJAZ11/Sl08g036660    CNLEFRLM
AtTIFY5a/AtJAZ8/AT1G30135       CDLELRLF
AtTIFY5b/AtJAZ7/At2g34600       CDLELRLL
OsTIFY5/Os07g05830              VELSLRLR
OsTIFY10c/Os09g26780            QGLGLGLR
OsTIFY11e/Os10g25230            DHLALAL-
AtTIFY11a/AtJAZ5/AT1G17380      KDLDLRL-
AtTIFY11b/AtJAZ6/AT1G72450      KNLELKL-
SlTIFY11a/SlJAZ3/Sl03g122190    RDLNLGIA
SlTIFY11b/SlJAZ4/Sl12g049400    NDLNLTIS
```

C

```
SlTIFY7/SlJAZ7/Solyc11g011030   FMDLNSKPK
AtTIFY11b/AtJAZ6/AT1G72450      IPDLNE-PT
AtTIFY11a/AtJAZ5/AT1G17380      LPDLNE-PT
SlTIFY11a/SlJAZ3/Sl03g122190    QLDLNFKL-
SlTIFY11b/SlJAZ4/Sl12g049400    HFDLNF---
```
