# Supplementary figures and images for: Identification of TIFY/JAZ family genes in Solanum lycopersicum and their regulation in response to abiotic stresses
Source: PLoS One. 2017 Jun 1;12(6):e0177381. doi: 10.1371/journal.pone.0177381 (PMC5453414; doi:10.1371/journal.pone.0177381)

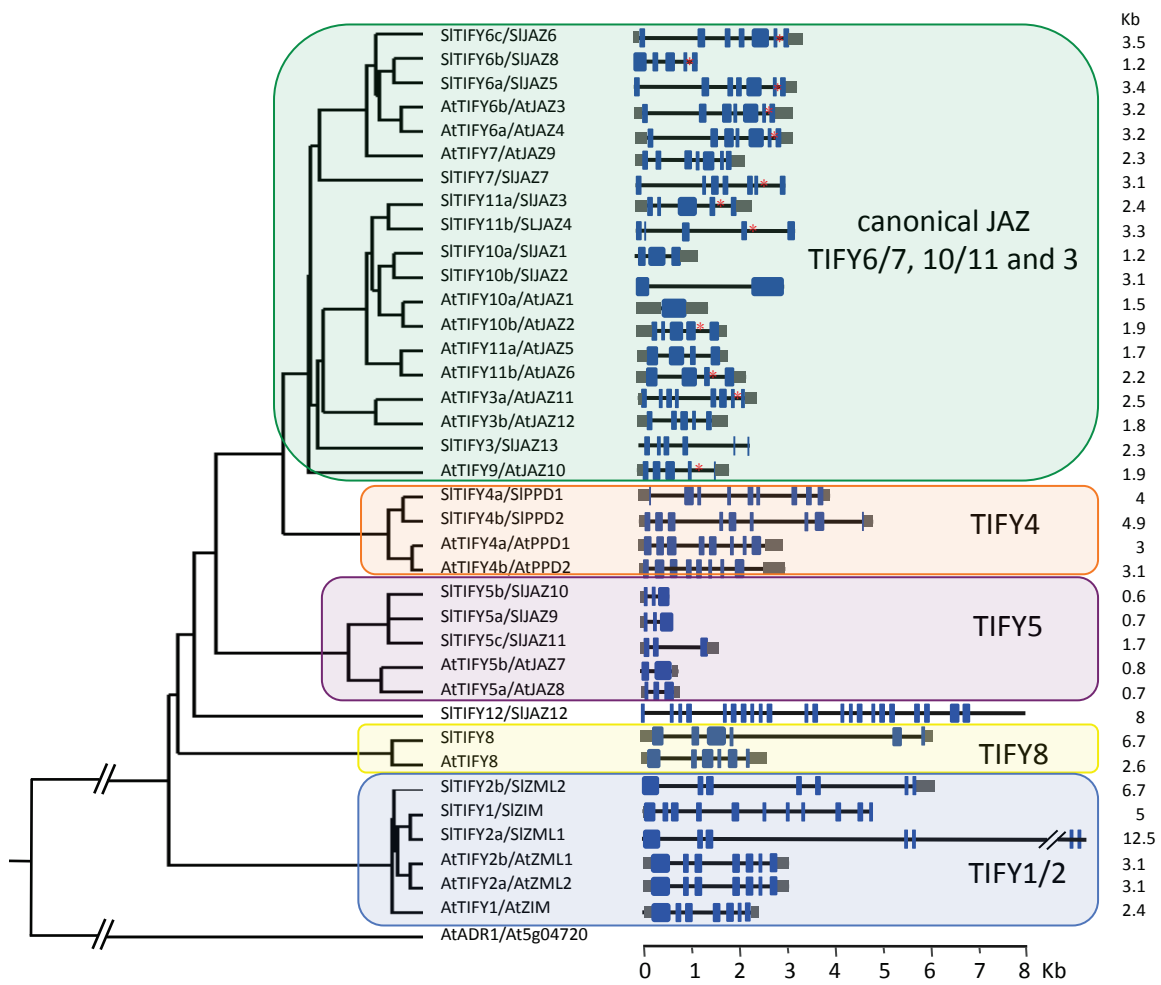

Supplement: S6 Fig — Different exon/intron structures of TIFY (JAZ, PPD and ZML) genes in S. lycopersicum and A. thaliana are shown. Blue boxes represent exons and black lines stand for introns. UTR regions are represented as grey boxes. Red asterisks represent Jas-intron retaining an in-frame stop codon. The length of the gene (in kilo bases) is annotated on the right and the scale is included at the bottom. (PDF) [file pone.0177381.s006.pdf]
