# Supplementary material for: Identification of TIFY/JAZ family genes in Solanum lycopersicum and their regulation in response to abiotic stresses
Source: PLoS One. 2017 Jun 1;12(6):e0177381. doi: 10.1371/journal.pone.0177381 (PMC5453414; doi:10.1371/journal.pone.0177381)
Supplement: S1 Table — (PDF) [file pone.0177381.s007.pdf]

Suppl. Table 1

Specific primers used in qPCR of *Solanum lycopersicum* cv. Glamour plants

Gene locus	Sequence Name	FW primer	RV primer
Solyc07g042170	*SlJAZ1*	TTCCCTCAAGGTGGAATGAAGGCT	TCCGAAACTCGGAACCACCAAATC
Solyc12g009220	*SlJAZ2*	ACCTGATCAACCAGAGAAGGCACA	ACCTGATCAACCAGAGAAGGCACA
Solyc03g122190	*SlJAZ3*	TTCCCTGCTGACAAAGCTAGAGCA	AGGGTGCAGATGAAACTGATCCGA
Solyc12g049400	*SlJAZ4*	GCCAAAGCCTCAGCAACAAAGGAT	ATCACTGCTCTGGCTTTCTCTGCT
Solyc03g118540	*SlJAZ5*	TCAGCTGTTCCGTCTAGCAGCATT	TGCATTTGGTGTAACAGGTGGTGC
Solyc01g005440	*SlJAZ6*	AGTCGATGCTGGTCTCAAACGTCA	TCGAAGACATTGACCATCCCACCA
Solyc11g011030	*SlJAZ7*	TTGCTATGGCTCGTAGAGCAACTC	TTTCCCAATGAACGCTTGACGACG
Solyc06g068930	*SlJAZ8*	TCGTCAACCTCCCAATCATAAC	GGAAAGGGTAGTGAGTGCATC
Solyc08g036640	*SlJAZ9*	TTTGGAGCTCACTCTTATGCCTCC	AGCTCAGTAGCATCGGAAACCACA
Solyc08g036620	*SlJAZ10*	GGAACTCACTCTTTCTCCTAGCAAC	TGGTGATGAAGGCTCAGACAGCTT
Solyc08g036660	*SlJAZ11*	GGAGTTTAGGCTTATGCCACCTTC	GGCTCAGATATTGGTGACAGACTC
Solyc01g009740	*SlJAZ12*	TGCGCATTCCGAGGCATGATGATA	CCTTCTTGCAATTGGCAACTCTGCT
Solyc08g006890	*SlTublin*	GGTGATGATGAAGCAGATGG	AAACAGACCGGCATTTTACAG
