# Supplementary material for: Identification of TIFY/JAZ family genes in Solanum lycopersicum and their regulation in response to abiotic stresses
Source: PLoS One. 2017 Jun 1;12(6):e0177381. doi: 10.1371/journal.pone.0177381 (PMC5453414; doi:10.1371/journal.pone.0177381)
Supplement: S2 Table — (PDF) [file pone.0177381.s008.pdf]

Supplemantary Table 2

In silico regulatory element analysis of *SlJAZ* gene promoters

	Abiotic stresses		Jasmonate responses					Defence responses		
Function of the element	Drought stress	ABA response	MeJA respons ive	MeJA response	Box-W1	WUN-moti	MYC2-box	ethylene response	Defense and stress	SA response
Name of the element	MBS	ABRE	TGACG motif	CGTCA motif	fungal elici RE	wound-motif	G-box CACGTG	ERE	TC-rich repeat	TCA-element
SlJAZ1/Sl07g042170		1	3				1		2	1
SlJAZ2/Sl12g009220		1						1	1	2
SlJAZ3/Sl03g122190	1	1	3	3					4	
SlJAZ4/Sl12g049400			1	1				1	2	
SlJAZ5/Sl03g118540	1					2			2	
SlJAZ6/Sl01g005440	1	7	1	1		1	3		2	
SlJAZ7/Sl11g011030						1		2	4	
SlJAZ8/Sl06g068930	1	1			2	2			1	
SlJAZ9/Sl08g036640	2	1							2	
SlJAZ10/Sl08g036620	2					1			1	
SlJAZ11/Sl08g036660	2	1	1					1		
SlJAZ12/Sl01g009740	2		2	2				1		1
SlTIFY8/Sl06g065650	1	1		3				1	3	4
SlZIM/Sl08g036640	2	2	1	1			1		2	
SlZML1/Sl10g047640	2		1	1				1	3	
SlZML2/Sl01g106030	2	1	2	2					2	
SlTIFY3/Sl01g103600	1	2	1	1	1	1			2	1
SlPPD1/Sl06g084120	1	2	3	2					2	2
SlPPD2/Sl09g065630		3							1	
