# Supplementary material for: Identification of TIFY/JAZ family genes in Solanum lycopersicum and their regulation in response to abiotic stresses
Source: PLoS One. 2017 Jun 1;12(6):e0177381. doi: 10.1371/journal.pone.0177381 (PMC5453414; doi:10.1371/journal.pone.0177381)
Supplement: S3 Table — (PDF) [file pone.0177381.s009.pdf]

Table S3. Original real-time quantitative qRT-PCR data of JA-treated tomato plants are shown as relative expression of *SlJAZ/SlTUB.*
Values are the average of three replicates; standard deviation is also reported. The log(10) transformation values are shown below.
Chini et al., PONE-D-17-04489
Identification of *TIFY/JAZ* family genes in *Solanum lycopersicum* and their regulation in response to abiotic stresses

Relative expression (average)

**root**	SlJAZ1	SlJAZ3	SlJAZ5	SlJAZ6	SlJAZ7	SlJAZ9	SlJAZ10	SlJAZ11
Control	1.00	1.02	1.00	1.00	1.00	1.01	1.00	1.00
12h	39.10	10.65	0.81	15.93	15.71	1.27	0.34	13.74
24h	38.98	7.85	0.53	12.41	17.80	1.31	0.25	21.26
48h	20.87	5.29	0.51	8.99	16.45	1.56	0.21	35.10
72h	13.64	2.80	0.49	8.55	4.64	2.62	0.20	31.27

**Leaves**	SlJAZ1	SlJAZ3	SlJAZ5	SlJAZ6	SlJAZ7	SlJAZ9	SlJAZ10	SlJAZ11
Control	1.00	1.09	1.00	1.00	1.00	1.00	1.00	1.00
12h	6.70	4.01	0.63	9.09	11.33	0.68	0.56	0.47
24h	4.70	3.97	0.56	4.24	10.08	0.39	0.60	0.45
48h	1.11	2.20	0.46	3.46	5.64	0.98	0.68	0.47
72h	1.10	1.16	0.34	3.29	5.27	1.04	0.71	0.44

Standard Deviation (Relative expression)

**root**	SlJAZ1	SlJAZ3	SlJAZ5	SlJAZ6	SlJAZ7	SlJAZ9	SlJAZ10	SlJAZ11
Control	0.06	0.25	0.02	0.06	0.08	0.15	0.08	0.06
12h	0.10	0.08	0.09	0.06	0.08	0.04	0.04	0.05
24h	0.18	0.07	0.06	0.04	0.18	0.05	0.06	0.07
48h	0.15	0.07	0.07	0.06	0.09	0.08	0.04	0.07
72h	0.08	0.08	0.07	0.07	0.07	0.06	0.06	0.06

**Leaves**	SlJAZ1	SlJAZ3	SlJAZ5	SlJAZ6	SlJAZ7	SlJAZ9	SlJAZ10	SlJAZ11
Control	0.07	0.56	0.09	0.09	0.08	0.04	0.04	0.08
12h	0.08	0.09	0.02	0.06	0.08	0.06	0.09	0.03
24h	0.06	0.07	0.02	0.06	0.18	0.08	0.04	0.01
48h	0.04	0.05	0.05	0.04	0.09	0.05	0.04	0.09
72h	0.07	0.04	0.08	0.06	0.07	0.03	0.08	0.09

log10-transform vales

**root**	SlJAZ1	SlJAZ3	SlJAZ5	SlJAZ6	SlJAZ7	SlJAZ9	SlJAZ10	SlJAZ11
0	0.00	0.01	0.00	0.00	0.00	0.00	0.00	0.00
12 h	1.59	1.03	-0.09	1.20	1.20	0.10	-0.47	1.14
24 h	1.59	0.90	-0.28	1.09	1.25	0.12	-0.60	1.33
48 h	1.32	0.72	-0.29	0.95	1.22	0.19	-0.68	1.55
72 h	1.13	0.45	-0.31	0.93	0.67	0.42	-0.69	1.50

**Leaves**	SlJAZ1	SlJAZ3	SlJAZ5	SlJAZ6	SlJAZ7	SlJAZ9	SlJAZ10	SlJAZ11
0	0.00	0.04	0.00	0.00	0.00	0.00	0.00	0.00
12 h	0.83	0.60	-0.20	0.96	1.05	-0.17	-0.25	-0.33
24 h	0.67	0.60	-0.26	0.63	1.00	-0.41	-0.22	-0.34
48 h	0.05	0.34	-0.34	0.54	0.75	-0.01	-0.17	-0.33
72 h	0.04	0.06	-0.47	0.52	0.72	0.02	-0.15	-0.36

Table S3. Original real-time quantitative qRT-PCR data of ABA-treated tomato plants are shown as relative expression of *SlJAZ/SlTUB.* Values are the average of three replicates; standard deviation is also reported. The log(10) transformation values are shown below.

Chini et al., PONE-D-17-04489

Identification of *TIFY/JAZ* family genes in *Solanum lycopersicum* and their regulation in response to abiotic stresses

Relative expression (average)

**root**	SlJAZ1	SlJAZ3	SlJAZ5	SlJAZ6	SlJAZ7	SlJAZ9	SlJAZ10	SlJAZ11
Control	1.00	1.00	1.00	1.01	1.00	1.03	1.00	1.00
12h	0.70	0.63	2.44	0.53	1.04	1.42	1.56	1.96
24h	0.66	0.63	4.87	0.48	1.61	1.44	1.65	2.88
48h	0.58	0.60	2.28	0.44	2.31	1.60	2.22	4.70
72h	0.53	0.32	1.88	0.32	1.18	4.71	3.07	4.27

**Leaves**	SlJAZ1	SlJAZ3	SlJAZ5	SlJAZ6	SlJAZ7	SlJAZ9	SlJAZ10	SlJAZ11
Control	1.00	1.00	1.00	1.00	1.00	1.00	1.00	1.00
12h	0.64	1.07	0.59	1.19	15.49	0.80	1.04	1.69
24h	0.32	0.96	0.49	0.90	17.16	1.39	1.52	1.82
48h	0.26	0.88	0.49	0.75	11.13	1.52	1.57	2.26
72h	0.26	0.31	0.45	0.72	7.09	0.61	2.90	4.99

Standard Deviation (Relative expression)

**root**	SlJAZ1	SlJAZ3	SlJAZ5	SlJAZ6	SlJAZ7	SlJAZ9	SlJAZ10	SlJAZ11
Control	0.06	0.07	0.07	0.05	0.08	0.08	0.03	0.06
12h	0.01	0.04	0.08	0.08	0.07	0.03	0.08	0.06
24h	0.03	0.09	0.05	0.05	0.06	0.09	0.07	0.04
48h	0.07	0.07	0.06	0.08	0.09	0.07	0.02	0.02
72h	0.05	0.05	0.07	0.06	0.09	0.03	0.02	0.04

**Leaves**	SlJAZ1	SlJAZ3	SlJAZ5	SlJAZ6	SlJAZ7	SlJAZ9	SlJAZ10	SlJAZ11
Control	0.08	0.08	0.03	0.07	0.07	0.03	0.04	0.07
12h	0.02	0.07	0.07	0.06	0.08	0.06	0.04	0.03
24h	0.06	0.08	0.03	0.03	0.04	0.04	0.03	0.05
48h	0.03	0.08	0.04	0.08	0.10	0.08	0.07	0.07
72h	0.06	0.03	0.04	0.09	0.07	0.07	0.07	0.08

log10-transform vales

**root**	SlJAZ1	SlJAZ3	SlJAZ5	SlJAZ6	SlJAZ7	SlJAZ9	SlJAZ10	SlJAZ11
0	0.00	0.00	0.00	0.00	0.00	0.01	0.00	0.00
12 h	-0.15	-0.20	0.39	-0.28	0.02	0.15	0.19	0.29
24 h	-0.18	-0.20	0.69	-0.32	0.21	0.16	0.22	0.46
48 h	-0.24	-0.22	0.36	-0.36	0.36	0.20	0.35	0.67
72 h	-0.28	-0.49	0.27	-0.49	0.07	0.67	0.49	0.63

**Leaves**	SlJAZ1	SlJAZ3	SlJAZ5	SlJAZ6	SlJAZ7	SlJAZ9	SlJAZ10	SlJAZ11
0	0.00	0.00	0.00	0.00	0.00	0.00	0.00	0.00
12 h	-0.20	0.03	-0.23	0.07	1.19	-0.10	0.02	0.23
24 h	-0.50	-0.02	-0.31	-0.04	1.23	0.14	0.18	0.26
48 h	-0.59	-0.05	-0.31	-0.13	1.05	0.18	0.19	0.35
72 h	-0.59	-0.51	-0.35	-0.14	0.85	-0.22	0.46	0.70

Table S3. Original real-time quantitative qRT-PCR data of NaCl-treated tomato plants are shown as relative expression of *SlJAZ/SlTUB.* Values are the average of three replicates; standard deviation is also reported. The log(10) transformation values are shown below.

Chini et al., PONE-D-17-04489

Identification of *TIFY/JAZ* family genes in *Solanum lycopersicum* and their regulation in response to abiotic stresses

Relative expression (average)

**root**	SlJAZ1	SlJAZ3	SlJAZ5	SlJAZ6	SlJAZ7	SlJAZ9	SlJAZ10	SlJAZ11
Control	1.00	1.00	1.00	1.00	1.00	1.03	1.00	1.00
12h	0.47	1.03	1.98	0.79	1.61	0.66	0.72	1.45
24h	0.26	0.59	1.88	0.63	1.72	0.69	0.62	1.45
48h	0.19	0.58	1.60	0.48	2.23	1.23	0.59	1.56
72h	0.14	0.26	1.35	0.30	2.46	1.62	0.53	1.63

**Leaves**	SlJAZ1	SlJAZ3	SlJAZ5	SlJAZ6	SlJAZ7	SlJAZ9	SlJAZ10	SlJAZ11
Control	1.00	1.00	1.00	1.00	1.00	1.00	1.00	1.00
12h	1.57	4.66	0.79	0.82	14.45	0.67	1.80	1.02
24h	0.33	3.30	0.71	0.42	9.78	0.66	1.96	1.05
48h	0.30	3.28	0.63	0.31	2.96	1.18	2.62	1.15
72h	0.16	0.87	0.54	0.21	2.83	1.41	2.48	1.46

Standard Deviation (Relative expression)

**root**	SlJAZ1	SlJAZ3	SlJAZ5	SlJAZ6	SlJAZ7	SlJAZ9	SlJAZ10	SlJAZ11
Control	0.06	0.07	0.09	0.06	0.08	0.08	0.03	0.06
12h	0.08	0.08	0.08	0.09	0.03	0.04	0.06	0.08
24h	0.01	0.02	0.05	0.01	0.06	0.08	0.08	0.04
48h	0.03	0.07	0.06	0.08	0.09	0.08	0.01	0.06
72h	0.09	0.05	0.06	0.03	0.05	0.08	0.02	0.04

**Leaves**	SlJAZ1	SlJAZ3	SlJAZ5	SlJAZ6	SlJAZ7	SlJAZ9	SlJAZ10	SlJAZ11
Control	0.08	0.08	0.03	0.09	0.07	0.03	0.04	0.07
12h	0.03	0.08	0.07	0.06	0.08	0.07	0.05	0.07
24h	0.02	0.05	0.03	0.05	0.02	0.03	0.04	0.06
48h	0.01	0.05	0.04	0.09	0.08	0.08	0.08	0.03
72h	0.02	0.05	0.04	0.01	0.09	0.07	0.06	0.08

log10-transform vale

**root**	SlJAZ1	SlJAZ3	SlJAZ5	SlJAZ6	SlJAZ7	SlJAZ9	SlJAZ10	SlJAZ11
0	0.00	0.00	0.00	0.00	0.00	0.01	0.00	0.00
12 h	-0.33	0.01	0.30	-0.10	0.21	-0.18	-0.15	0.16
24 h	-0.58	-0.23	0.27	-0.20	0.23	-0.16	-0.21	0.16
48 h	-0.73	-0.24	0.20	-0.32	0.35	0.09	-0.23	0.19
72 h	-0.86	-0.59	0.13	-0.52	0.39	0.21	-0.28	0.21

**Leaves**	SlJAZ1	SlJAZ3	SlJAZ5	SlJAZ6	SlJAZ7	SlJAZ9	SlJAZ10	SlJAZ11
0	0.00	0.00	0.00	0.00	0.00	0.00	0.00	0.00
12 h	0.20	0.67	-0.10	-0.09	1.16	-0.18	0.25	0.01
24 h	-0.48	0.52	-0.15	-0.37	0.99	-0.18	0.29	0.02
48 h	-0.52	0.52	-0.20	-0.50	0.47	0.07	0.42	0.06
72 h	-0.81	-0.06	-0.27	-0.68	0.45	0.15	0.39	0.16

Table S3. Original real-time quantitative qRT-PCR data of mannitol-treated tomato plants are shown as relative expression of *SlJAZ/SlTUB*. Values are the average of three replicates; standard deviation is also reported. The log(10) transformation values are shown below.

Chini et al., PONE-D-17-04489

Identification of *TIFY/JAZ* family genes in *Solanum lycopersicum* and their regulation in response to abiotic stresses

Relative expression (average)

**root**	SlJAZ1	SlJAZ3	SlJAZ5	SlJAZ6	SlJAZ7	SlJAZ9	SlJAZ10	SlJAZ11
Control	1.00	1.00	1.00	1.00	1.00	1.03	1.00	1.00
12h	0.53	0.46	0.56	0.86	1.73	0.36	0.31	1.75
24h	0.21	0.37	0.48	0.73	1.74	0.37	0.39	0.82
48h	0.15	0.31	0.85	0.48	1.72	0.82	0.39	0.82
72h	0.13	0.19	0.93	0.25	1.76	0.84	0.21	0.73

**Leaves**	SlJAZ1	SlJAZ3	SlJAZ5	SlJAZ6	SlJAZ7	SlJAZ9	SlJAZ10	SlJAZ11
Control	1.00	1.00	1.00	1.00	1.00	1.00	1.00	1.00
12h	1.02	0.84	2.51	1.12	2.28	2.48	0.55	0.38
24h	0.39	0.79	1.73	1.12	2.75	1.94	0.35	0.39
48h	0.36	0.36	0.59	1.10	7.62	1.85	0.32	0.66
72h	0.33	0.32	0.28	1.11	6.99	1.38	0.24	0.77

Standard Deviation (Relative expression)

**root**	SlJAZ1	SlJAZ3	SlJAZ5	SlJAZ6	SlJAZ7	SlJAZ9	SlJAZ10	SlJAZ11
Control	0.06	0.07	0.09	0.06	0.08	0.08	0.03	0.06
12h	0.02	0.06	0.03	0.02	0.05	0.06	0.07	0.03
24h	0.03	0.09	0.05	0.05	0.04	0.09	0.09	0.02
48h	0.03	0.09	0.03	0.01	0.05	0.02	0.01	0.03
72h	0.05	0.03	0.04	0.02	0.05	0.09	0.04	0.01

**Leaves**	SlJAZ1	SlJAZ3	SlJAZ5	SlJAZ6	SlJAZ7	SlJAZ9	SlJAZ10	SlJAZ11
Control	0.08	0.08	0.03	0.09	0.07	0.03	0.04	0.07
12h	0.08	0.04	0.07	0.03	0.03	0.04	0.05	0.02
24h	0.04	0.08	0.03	0.05	0.06	0.05	0.02	0.04
48h	0.03	0.07	0.09	0.03	0.06	0.07	0.02	0.01
72h	0.09	0.07	0.03	0.05	0.06	0.08	0.04	0.04

log10-transform vales

**root**	SlJAZ1	SlJAZ3	SlJAZ5	SlJAZ6	SlJAZ7	SlJAZ9	SlJAZ10	SlJAZ11
0	0.00	0.00	0.00	0.00	0.00	0.01	0.00	0.00
12 h	-0.27	-0.34	-0.25	-0.07	0.24	-0.44	-0.52	0.24
24 h	-0.69	-0.44	-0.32	-0.14	0.24	-0.43	-0.41	-0.09
48 h	-0.82	-0.51	-0.07	-0.32	0.24	-0.08	-0.41	-0.09
72 h	-0.87	-0.71	-0.03	-0.60	0.24	-0.08	-0.67	-0.14

**Leaves**	SlJAZ1	SlJAZ3	SlJAZ5	SlJAZ6	SlJAZ7	SlJAZ9	SlJAZ10	SlJAZ11
0	0.00	0.00	0.00	0.00	0.00	0.00	0.00	0.00
12 h	0.01	-0.08	0.40	0.05	0.36	0.39	-0.26	-0.42
24 h	-0.41	-0.10	0.24	0.05	0.44	0.29	-0.46	-0.41
48 h	-0.44	-0.44	-0.23	0.04	0.88	0.27	-0.49	-0.18
72 h	-0.49	-0.49	-0.56	0.04	0.84	0.14	-0.63	-0.11
